# All-electrical switching of a topological non-collinear antiferromagnet at room temperature

**DOI:** 10.1093/nsr/nwac154

**Published:** 2022-08-04

**Authors:** Yongcheng Deng, Xionghua Liu, Yiyuan Chen, Zongzheng Du, Nai Jiang, Chao Shen, Enze Zhang, Houzhi Zheng, Hai-Zhou Lu, Kaiyou Wang

**Affiliations:** State Key Laboratory for Superlattices and Microstructures, Institute of Semiconductors, Chinese Academy of Sciences, Beijing 100083, China; Center of Materials Science and Optoelectronics Engineering, University of Chinese Academy of Sciences, Beijing 100049, China; State Key Laboratory for Superlattices and Microstructures, Institute of Semiconductors, Chinese Academy of Sciences, Beijing 100083, China; Center of Materials Science and Optoelectronics Engineering, University of Chinese Academy of Sciences, Beijing 100049, China; Institute for Quantum Science and Engineering and Department of Physics, Southern University of Science and Technology (SUSTech), Shenzhen 518055, China; International Quantum Academy, Shenzhen 518048, China; Institute for Quantum Science and Engineering and Department of Physics, Southern University of Science and Technology (SUSTech), Shenzhen 518055, China; International Quantum Academy, Shenzhen 518048, China; State Key Laboratory for Superlattices and Microstructures, Institute of Semiconductors, Chinese Academy of Sciences, Beijing 100083, China; Center of Materials Science and Optoelectronics Engineering, University of Chinese Academy of Sciences, Beijing 100049, China; State Key Laboratory for Superlattices and Microstructures, Institute of Semiconductors, Chinese Academy of Sciences, Beijing 100083, China; Center of Materials Science and Optoelectronics Engineering, University of Chinese Academy of Sciences, Beijing 100049, China; State Key Laboratory for Superlattices and Microstructures, Institute of Semiconductors, Chinese Academy of Sciences, Beijing 100083, China; Center of Materials Science and Optoelectronics Engineering, University of Chinese Academy of Sciences, Beijing 100049, China; State Key Laboratory for Superlattices and Microstructures, Institute of Semiconductors, Chinese Academy of Sciences, Beijing 100083, China; Center of Materials Science and Optoelectronics Engineering, University of Chinese Academy of Sciences, Beijing 100049, China; Institute for Quantum Science and Engineering and Department of Physics, Southern University of Science and Technology (SUSTech), Shenzhen 518055, China; International Quantum Academy, Shenzhen 518048, China; State Key Laboratory for Superlattices and Microstructures, Institute of Semiconductors, Chinese Academy of Sciences, Beijing 100083, China; Center of Materials Science and Optoelectronics Engineering, University of Chinese Academy of Sciences, Beijing 100049, China; Beijing Academy of Quantum Information Sciences, Beijing 100193, China; Center for Excellence in Topological Quantum Computation, University of Chinese Academy of Sciences, Beijing 100049, China

**Keywords:** spintronics, non-collinear antiferromagnetic Weyl semimetals, all-electrical switching, spin-orbit torques

## Abstract

Non-collinear antiferromagnetic Weyl semimetals, combining the advantages of a zero stray field and ultrafast spin dynamics, as well as a large anomalous Hall effect and the chiral anomaly of Weyl fermions, have attracted extensive interest. However, the all-electrical control of such systems at room temperature, a crucial step toward practical application, has not been reported. Here, using a small writing current density of around 5 × 10^6^ A·cm^–2^, we realize the all-electrical current-induced deterministic switching of the non-collinear antiferromagnet Mn_3_Sn, with a strong readout signal at room temperature in the Si/SiO_2_/Mn_3_Sn/AlO_x_ structure, and without external magnetic field or injected spin current. Our simulations reveal that the switching originates from the current-induced intrinsic non-collinear spin-orbit torques in Mn_3_Sn itself. Our findings pave the way for the development of topological antiferromagnetic spintronics.

## INTRODUCTION

Antiferromagnets have recently attracted tremendous interest as candidates for next-generation spintronics devices, as they have the prospect of offering higher storage density and faster data processing than their ferromagnetic counterparts [1,2]. However, the weak readout signals of conventional collinear antiferromagnets driven by electrical approaches greatly restrict their practical applications [3,4]. Alternatively, large magnetotransport signatures, such as the intrinsic anomalous Hall effect in topological antiferromagnets, could provide a solution to this issue [5,6]. In particular, the non-collinear antiferromagnetic Weyl semimetal Mn_3_Sn has recently fascinated the condensed matter physics and information technology communities because of its non-trivial band topology [7,8] and unusual magnetic responses [9–11].

**Figure 1.**
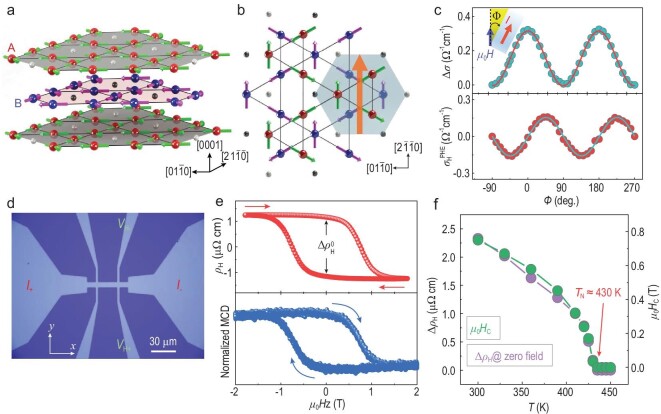
Crystal and magnetic structures, and the magnetotransport and magnetic properties of the Mn_3_Sn device. (a) Mn_3_Sn crystal structure. The large red and blue spheres (small gray and black spheres) represent the Mn (Sn) atoms at the *z* = 0 and 1/2 planes, respectively. (b) Magnetic structure of Mn_3_Sn. The magnetic moments of Mn (green and pink arrows in different layers) are arranged along the kagome planes and form a spin structure with inverse triangular texture. The six adjacent moments between layers (light blue hexagon) constitute ferroic ordering of a cluster magnetic octupole (large orange arrow). (c) Angular dependence of the in-plane longitudinal magnetoconductivity $\Delta \sigma \ $(top) and planar Hall conductivity $\sigma _{\rm{H}}^{{\rm{PHE}}}$ (bottom) of the 50-nm-thick Mn_3_Sn device at room temperature and 1.8 T. The red and cyan solid lines for $\Delta \sigma $ and $\sigma _{\rm{H}}^{{\rm{PHE}}}$ are the fitting results using the theoretical equations for the chiral anomaly (Equations S6 and S7 in Supplementary Section S6). (d) Optical micrograph of our fabricated Hall device and measurement scheme. (e) Anomalous Hall resistivity (top) and magnetic circular dichroism (MCD) signal (bottom) versus ${\mu }_0{H}_{\rm{Z}}$ for the 50-nm-thick Mn_3_Sn device at room temperature. Both of them exhibit clear hysteresis loops with a coercive field ${\mu }_0{H}_{\rm{C}}$ of ∼0.75 T. (f) Temperature dependence of $\Delta \rho _{\rm{H}}^0$ and ${\mu }_0{H}_{\rm{C}}$ derived from the hysteresis loops of the 50-nm-thick Mn_3_Sn device, suggesting a Néel temperature *T*_N_ ≈ 430 K (Supplementary Fig. S7).

Mn_3_Sn hosts an ABAB stacking sequence of the (0001) kagome lattice of Mn (Fig. 1a), with a 120° non-collinear antiferromagnetic ordering of the Mn magnetic moments below the Néel temperature of *T*_N_ ≈ 430 K [9]. This antiferromagnetic state on the kagome bilayers can be viewed as a ferroic ordering of a cluster magnetic octupole (Fig. 1b), which macroscopically breaks the time-reversal symmetry and results in a large anomalous Hall effect [9,12]. With the assistance of an auxiliary magnetic field, the deterministic switching of the magnetic octupole in Mn_3_Sn has been achieved by spin-orbit torques from a heavy-metal layer [13,14]. However, as a critical step toward practical application, the field-free manipulation of Mn_3_Sn, driven by electrical currents at room temperature, has not been reported. Here, we demonstrate the all-electrical switching of topological antiferromagnetic states in heavy-metal-layer-free Mn_3_Sn devices.

## RESULTS

Experiments were performed on sputter-deposited Mn_3_Sn (50 nm)/AlO_x_ (2 nm) thin films, and on the reference samples with heavy metals consisting of Ru (3 nm)/Mn_3_Sn (50 nm)/AlO_x_ (2 nm) and Ru (3 nm)/Mn_3_Sn (50 nm)/Pt (8 nm)/AlO_x_ (2 nm). All samples were deposited on thermally oxidized Si substrate. Unless otherwise stated, all the measurements were performed on the Mn_3_Sn (50 nm)/AlO_x_ (2 nm) materials or devices without heavy metals. We first characterize the structure, transport properties and magnetic properties of Mn_3_Sn. The X-ray diffraction peaks of (010) and (020) at 18° and 36° confirm the hexagonal D0_19_ Mn_3_Sn structure, and no additional peaks coming from plausible impurity phases were observed [15,16] (see the details in Supplementary Section S1 and more related analysis in Supplementary Sections S2–S4). The microstructure of our film and the chemical composition of Mn_3.06_Sn_0.94_ were measured by cross-sectional high-resolution transmission electron microscopy (HR-TEM) and energy-dispersive X-ray spectroscopy (EDX) [17,18] (Supplementary Fig. S3), respectively. The magnetotransport phenomena observed in our thin films (Fig. 1c and Supplementary Fig. S4) are consistent with previous measurements [13], where the angular dependence of the in-plane longitudinal magnetoconductivity $( {{\rm{\Delta }}\sigma \ = \ \sigma - {\sigma }_ \bot } )\ $and planar Hall conductivity $\sigma _{\rm{H}}^{{\rm{PHE}}}$ can be well fitted by the theoretical equations for the chiral anomaly of Weyl fermions [19] (Fig. 1c and Supplementary Section S6).

We measured the anomalous Hall resistivity ${\rho }_{\rm{H}}$ as a function of the out-of-plane magnetic field ${\mu }_0{H}_{\rm{Z}}$ to quantitatively estimate the population of switchable domains in the device (Fig. 1d). A clear hysteresis of the anomalous Hall resistivity with a zero-field change $\Delta \rho _{\rm{H}}^0$ is observed (Fig. 1e (top)), which shows that the negative (positive) value of ${\rho }_{\rm{H}}$ is produced by the ‘+*z* (−*z*) domain’ with the positive +*z* (negative −*z*) component of the polarization direction of the magnetic octupole [13,15,20,21]. The field-swept measurements of the magnetic circular dichroism (MCD) exhibit a hysteresis loop of *M − *${\mu }_0{H}_{\rm{Z}}$ with a coercive field of ${\mu }_0{H}_{\rm{C}}$$\approx {\rm{\ 0}}{\rm{.75\ T}}$ (Fig. 1e (bottom)), which is in agreement with the measured anomalous Hall resistivity (Fig. 1e (top)). Moreover, the *M − *${\mu }_0{H}_{\rm{Z}}\ $curves were measured at different temperatures (*T*) using a vibrating sample magnetometer and the extracted magnetization of ∼8 emu/cc at 300 K and 5 mT (Supplementary Fig. S5f), which is comparable with previous reported values [13,15,20,21]. The longitudinal resistivity and Hall resistivity as a function of temperature under zero magnetic field are illustrated in Supplementary Fig. S6. Both the *M* − *T* and ${\rho }_{{\rm{H\ }}}$ − *T* curves show a rapid decrease at ∼250 K, which corresponds to the transition to spiral phase [22–24]. The Néel temperature *T*_N_, corresponding to the disappearance of $\Delta \rho _{\rm{H}}^0$ and ${\mu }_0{H}_{\rm{C}}$ of the anomalous Hall hysteresis loops, is found to be ∼430 K (Fig. 1f and Supplementary Fig. S7), which is close to that of single crystal Mn_3_Sn [9]. The results confirm that our thin films have physical properties similar to those of previous reports.

**Figure 2.**
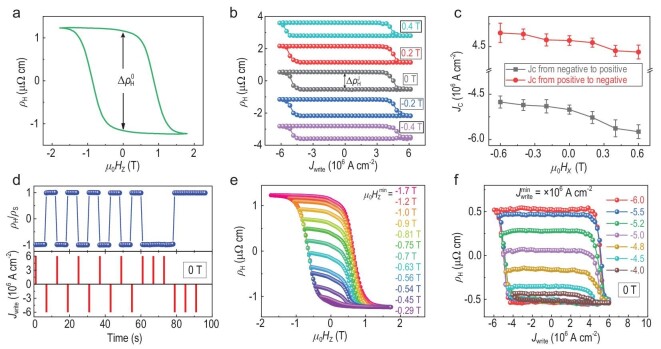
Zero-field current-induced switching of the antiferromagnetic states in the Mn_3_Sn device. (a) Anomalous Hall resistivity ${\rho }_{\rm{H}}$ dependence on ${\mu }_0{H}_Z$ for the Mn_3_Sn device at room temperature. (b) ${\rho }_{\rm{H}}$ versus ${J}_{{\rm{write}}}$ at ${\mu }_0{H}_x$ = 0, ±0.2 T, ±0.4 T for the Mn_3_Sn device at room temperature. (c) The critical current density ${J}_{\rm{c}}$ as a function of ${\mu }_0{H}_x$ for positive and negative current-sweeping directions, respectively, of the ${\rho }_{\rm{H}}$ vs. ${J}_{{\rm{write}}}$ loops. (d) The normalized ${\rho }_{\rm{H}}$ (top) and ${J}_{{\rm{write}}}$ (bottom) for the Mn_3_Sn device at room temperature for a series of applied positive and negative ${J}_{{\rm{write}}}$ = 5 × 10^6^ A·cm^–2^, where ${\rho }_{\rm{H}}$ was measured at ${I}_{{\rm{read}}} = {\rm{\ 0}}{\rm{.1}}$ mA after each writing current pulse. (e and f) ${\rho }_{\rm{H}}$ versus ${\mu }_0{H}_{\rm{Z}}$ and ${J}_{{\rm{write}}}$ loops for the Mn_3_Sn device at room temperature. The minimum ${\mu }_0H_{\rm{Z}}^{{\rm{min}}}$ and $J_{{\rm{write}}}^{{\rm{min}}}$ determine the magnitude of the field- and current-driven Hall resistivity switching.

We then examined the possible current-induced topological non-collinear antiferromagnetic state switching. For the reference samples, the current-induced deterministic switching can only be observed under an auxiliary magnetic field for Ru/Mn_3_Sn/Pt devices (Supplementary Fig. S8), which is consistent with previous works [13,14]. Interestingly, different current-induced switching behaviors were observed in our heavy-metal-layer-free devices. Figure 2a presents the ${\rho }_{\rm{H}}{\rm{ - }}{\mu }_0{H}_{\rm{Z}}$ curve of a 50-nm-thick Mn_3_Sn device. Under zero magnetic field, a 50-ms writing current pulse ${I}_{{\rm{write}}}$ followed by a DC reading current of ${I}_{{\rm{read}}}{\rm{ = \ 0}}{\rm{.1\ mA}}$ is applied along the *x* direction. Surprisingly, as shown by the black curve in Fig. 2b, the electrical current flowing through the device leads to a clear negative (positive) jump in ${\rho }_{\rm{H}}$ at a positive (negative) threshold writing current, implying a reversing of the *z* component of the octupole. The magnitude of the Hall resistivity jump $\Delta \rho _{\rm{H}}^{\rm{J}}$ is ∼58% of $\Delta \rho _{\rm{H}}^0$ in the field-swept measurements (Fig. 2a). Figure 2b also shows the ${\rho }_{\rm{H}}-{J}_{{\rm{write}}}$ loops in an in-plane magnetic field ${\mu }_0{H}_x$ of ±0.2 T and ±0.4 T. With the increase of positive (negative) applied magnetic field, the gradual shift of ${\rho }_{\rm{H}}-{J}_{{\rm{write}}}$ loops toward a negative (positive)$\ {J}_{{\rm{write}}}$, together with the reduction of $\Delta \rho _{\rm{H}}^{\rm{J}}$, is observed, which is clearer in the field dependence of the threshold current in Fig. 2c. The current-induced magnetization switching disappears for a sufficiently large bias field, e.g. ${\rm{1\ T\ > }}\ {\mu }_0{H}_{\rm{C}}$, probably because the magnetic octupoles are aligned to the external field direction.

Compared with the Mn_3_Sn/(heavy-) metal reference devices (Supplementary Fig. S8), here, the writing current is fully injected into the Mn_3_Sn layer without passing through a highly conductive metal layer. In addition, the strong inversion asymmetry along the $x( y )$ direction was confirmed by our non-linear Hall measurements [25,26]. As a low frequency AC current is applied along the *x*(*y*) direction, a considerable second harmonic voltage is measured along the $y( x )$ direction in our Mn_3_Sn device, probably due to the boundaries between grains and/or magnetic domains. The non-linear Hall effect in our Mn_3_Sn device is considerably stronger than that measured in the reference samples, justifying a larger Rashba effect in the heavy-metal-layer-free device (Supplementary Fig. S9). We explain that the field-free deterministic switching originates from current-induced spin accumulations in the Mn_3_Sn layer without inversion symmetry.

Notably, the critical writing current density ${J}_{\rm{c}}$ required for switching the octupole, at zero magnetic field, is estimated to be 5 × 10^6^ A·cm^–2^ in our Mn_3_Sn device, which is less than half that required for the reference Ru/Mn_3_Sn/Pt/AlO_x_ sample. This is nearly one order of magnitude smaller than the recently reported value 4 × 10^7^ A·cm^–2^ for the collinear antiferromagnet/Pt devices [4], and comparable to the values reported for antiferromagnet/ferromagnet [27] and collinear antiferromagnet devices [3,28]. The increase of the device temperature due to Joule heating at the maximum current pulse is estimated to be 38 K (Supplementary Fig. S10). The temperature of the device is still substantially lower than the Néel temperature (≈430 K) even when ${J}_{{\rm{write}}}$ is on, thus the Joule heating effect cannot be the predominant reason behind the observed switching. Furthermore, we performed the same zero-field current-induced switching measurements at lower temperatures (200 K and 250 K), which show similar behaviors of switching with slightly larger threshold currents (Supplementary Fig. S11).

Our experiments confirm that the deterministic switching of the Mn_3_Sn devices is due to the current-induced torque exerted on the non-collinear antiferromagnetic spin texture. This reproducible bipolar switching can act as an antiferromagnetic memory. The alternating current pulses ($|{J}_{{\rm{write}}}| {\rm{\ }} \rangle {J}_{\rm{c}}$) along opposite directions switch the antiferromagnetic state back and forth reproducibly (Fig. 2d), indicating its reliable controllability. Remarkably, our Mn_3_Sn device can provide multilevel signals by varying the magnitude of ${\mu }_0{H}_{\rm{Z}}$ or ${J}_{{\rm{write}}}$. With a fixed maximum positive magnetic field (Fig. 2e) or writing current (Fig. 2f), the change of ${\rho }_{\rm{H}}$ increases with the increasing magnitude of ${\mu }_0H_{\rm{Z}}^{{\rm{min}}}$ or $J_{{\rm{write}}}^{{\rm{min}}}$ (Magnetic and magnetotransport measurements in Methods). This multilevel plasticity controlled by electrical currents at zero magnetic field suggests the great potential of Mn_3_Sn in neuromorphic computing [21,27,29,30].

**Figure 3.**
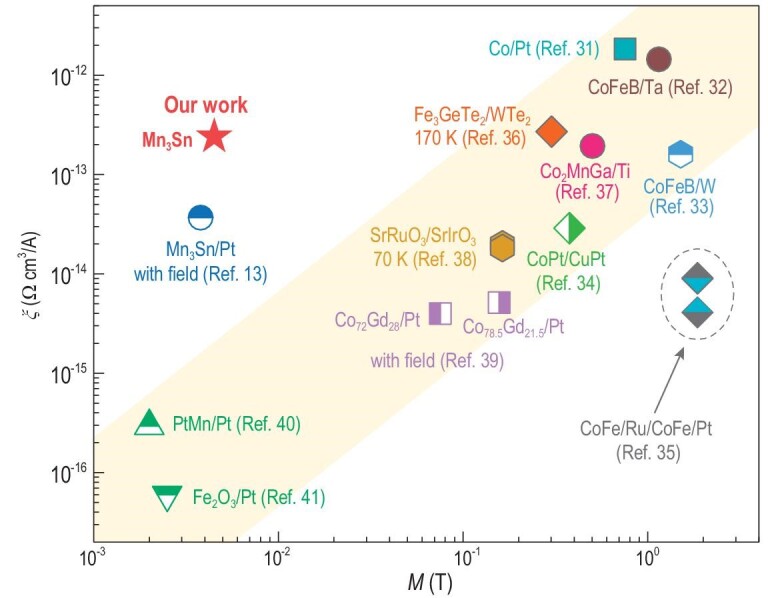
Readout efficiency as a function of magnetization. Double-logarithmic plot of the readout efficiency $\xi \ = {\rm{\ }}\Delta \rho _{\rm{H}}^{\rm{J}}/{J}_{\rm{c}}$ as a function of the magnetization *M* for various materials, including ferromagnets (Co/Pt [31], CoFeB/Ta [32], CoFeB/W [33], CoPt/CuPt [34], CoFe/Ru/CoFe/Pt [35], Fe_3_GeTe_2_/WTe_2_ [36], Co_2_MnGa/Ti [37] and SrRuO_3_/SrIrO_3_ [38]), ferrimagnets (Co_x_Gd_1-x_/Pt [39]), collinear antiferromagnets (PtMn/Pt [40] and Fe_2_O_3_/Pt [41]) and Mn_3_Sn/Pt [13], at various temperatures. The red star indicates our Mn_3_Sn device at room temperature. The yellow shaded region highlights the scaling law of $\xi $ with$\ M$.

To compare the efficiency of the readout signal driven by an electrical current with that of other heavy-metal/magnet bilayer structures, we defined the readout efficiency as $\xi \ = {\rm{\ }}\Delta \rho _{\rm{H}}^{\rm{J}}/{J}_{\rm{c}}$. In the case of our Mn_3_Sn device, $\xi $ is ∼2.4 × 10^–13^ Ω·cm^3^/A (marked with a red star in Fig. 3), which is close to readout efficiencies of ferromagnetic materials such as Co_2_MnGa and CoFeB [31–38] but one to three orders of magnitude larger than those of ferrimagnets, collinear antiferromagnets and Mn_3_Sn/Pt devices [13,39–41]. Interestingly, a scaling law of $\xi $ with the magnetization *M* is observed for collinear antiferromagnets, ferrimagnets and ferromagnets, as indicated by the shaded region in Fig. 3. This is because the anomalous Hall resistance is proportional to the magnetization, whereas the critical switching current is insensitive to the magnetization in the case of the current-induced magnetization switching dominated by the spin Hall effect in heavy-metal/magnet bilayer systems [42]. The large $\xi $ for Mn_3_Sn is due to the strong anomalous Hall effect originating from the non-zero Berry curvature in momentum space [7,8]. The readout efficiency obtained in our pure Mn_3_Sn is one order of magnitude higher than that in ref. [13], probably because our Mn_3_Sn devices have higher inversion asymmetry and do not have a heavy-metal layer. We can thus achieve a strong readout anomalous Hall signal driven by a small writing current of 5 × 10^6^ A·cm^–2^ in our Mn_3_Sn device.

## DISCUSSION

Now we illustrate the possible mechanisms of intrinsic non-collinear spin-orbit torques that induce the deterministic all-electrical switching in our Mn_3_Sn device. We argue that the current-induced switching requires an inversion asymmetry in our polycrystal device, for the following reasons.

**Figure 4.**
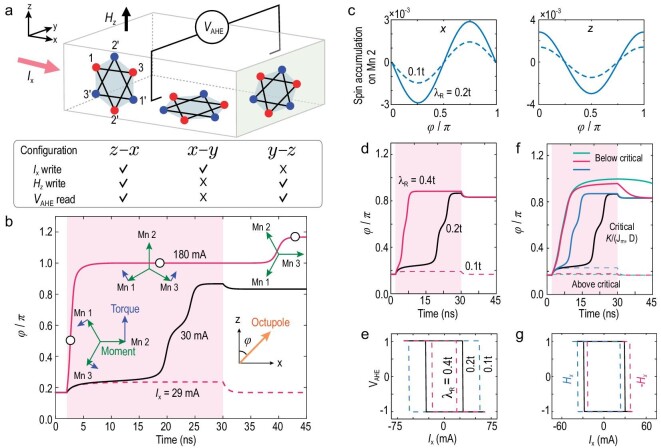
Numerically simulated switching of the Mn_3_Sn octupole. The spin-orbit coupling converts the current ${I}_x$ (experimental ${J}_{{\rm{write}}}$) into spin accumulations, which exert intrinsic non-collinear spin-orbit torques to rotate the Mn moments and octupole. (a) The kagome lattices in polycrystal can be decomposed into three configurations, *z*–*x, x*–*y* and *y*–*z*. ${I}_x$ can switch configurations *z*–*x* and *x*–*y* and ${V}_{{\rm{AHE}}}$ can read out configurations *z*–*x* and *y*–*z*, so the simulation focuses on configuration *z*–*x*. (b) Simulated octupole angle $\varphi $ versus time, driven by a ${I}_x$ pulse with duration of 2–30 ns (pink shadowed region), for ${I}_x$ below (29 mA), at (30 mA), and well above (180 mA) the critical current. For ${I}_x$= 180 mA, the insets show the dynamics of the torques (blue arrows) being exerted on the Mn moments (green arrows), at three stages (hollow circles), as $\varphi $ starts from $\pi / 6$ (an easy axis), then is switched to $\pi $ (normal to ${I}_x$) after turning on ${I}_x$, and finally relaxes to $7\pi / 6$ (another easy axis) after turning off ${I}_x$. (c) Simulated *x*- and *z*-direction spin accumulations on Mn 2 (denoted in panel (a)) as a function of $\varphi $, for different Rashba spin-orbit coupling ${\lambda }_R$ (Equation 2 in Methods of Supplementary). (d) The same as the 30 mA case in panel (b), but also for ${\lambda }_R$ below and above the critical value. (e) Simulated hysteresis loops of the Hall response ${V}_{{\rm{AHE}}}$ driven by ${I}_x$, for different ${\lambda }_R$. (f) The same as the 30 mA case in panel (b). Combinations of the magnetic-structure parameters $K/( {{J}_m,D} )$ (Equation 1 in Methods of Supplementar) below (solid curves) and above (dashed curves) the critical combination are also considered. (g) The same as the ${\lambda }_R = {\rm{\ }}0.2t$ case in panel (e). The presence of the *x*-axis magnetic field ${H}_x$ is also considered. More simulation results by changing ${\lambda }_R$, $K/( {{J}_m,D} )$, and initial states can be found in Supplementary Section S16.

In our Mn_3_Sn polycrystal, the measured non-zero anomalous Hall signal implies that the different configurations in our samples are not compensated. To understand this, we note that any crystal grain in the polycrystalline Mn_3_Sn can be decomposed into the *z*–*x, x*–*y* and *y*–*z* configurations, according to the direction of kagome plane (Fig. 4a). The octupole rotates only in the kagome plane, so the out-of-plane magnetic field ${H}_z$ can only switch configurations *z*–*x* and *y*–*z*. Considering the weak tunneling between the kagome layers, ${I}_x$ is not expected to be able to switch configuration *y*–*z* effectively, i.e. the in-plane current ${I}_x$ can only switch configurations *z*–*x* and *x*–*y*. The anomalous Hall signal depends on the *z* component of the octupole, so ${V}_{{\rm{AHE}}}$ can be used to read out only configurations *z*–*x* and *y*–*z*. This infers that the Hall response of the ${\mu }_0{H}_z$-hysteresis is around 2 to 2.3 times (considering that the octupole may relax at positions within ±30 degrees from the full polarization) that of the ${J}_{{\rm{write}}}$-hysteresis, close to the data in Fig. 2a and b. According to the above discussion, only configuration *z*–*x* in Fig. 4a can be switched by the current and is measurable by the anomalous Hall effect.

More importantly, the *z*–*x* configuration of single-crystal Mn_3_Sn requires an inversion asymmetry to be deterministically switched. Because the octupole is a pseudo vector, which is invariant under inversion, it does not reverse as the current changes sign, if there is inversion symmetry (see also the symmetry and microscopic analysis in Supplementary Sections S13 and S14). The occurrence of the inversion asymmetry in polycrystalline Mn_3_Sn was confirmed by our non-linear Hall measurements. The inversion asymmetry can induce Rashba-like spin-orbit coupling, which can convert the injected electric current into spin currents or spin accumulations. The spin accumulations, when not aligned with the local Mn moments, can induce intrinsic non-collinear spin-orbit torques to rotate the Mn moments. The octupole is defined by the Mn moments in the Mn_3_Sn magnetic structure with inverse triangular spin structure (Supplementary Sections S15). Thus, in this sense, it is switchable by the current, and its *z* component leads to a measurable anomalous Hall signal.

To verify our speculations regarding the microscopic mechanism behind the observed switching, we performed numerical simulations for the octupole polarization. For a given electrical current ${I}_x$, the simulation starts with an initial magnetic structure of the Mn moments ${{{\bf m}}}_{ia}$, which behave as local Zeeman fields on the electrons described by an s–d model. Using the linear-response theory, the local spin accumulations induced by the current in the presence of the Rashba spin-orbit coupling are calculated. The effective magnetic field of the spin accumulations is then converted into magnetic torques ${{{\bf T}}}_{ia}$ in the Landau-Lifshitz-Gilbert equation to generate a new magnetic structure. The above steps are iterated until the magnetic structure converges to yield the octupole moment for the given injected current (see details in Methods Section simulating current-induced switching of the non-collinear antiferromagnet).

Figure 4b shows the simulated octuple angle $\varphi $ as a function of time at different current intensities, which demonstrates that the current must be sufficiently strong to drive a switching. Its insets also show the microscopic dynamics in terms of the calculated torques being exerted on the Mn moments at different stages of a successful switching for ${I}_x$, well above the critical value. The non-collinear antiferromagnetic structure tends to be maintained during the switching, and the rotation of the Mn moments (collectively as octupole) is determined by the sum of the nutation tendencies of the sublattice moments driven by the torques. Figure 4c–e shows that a larger Rashba spin-orbit coupling ${\lambda }_R$ leads to an enhanced spin accumulation, faster switching and a smaller critical current. Figure 4f shows that increasing/decreasing the magnetic-structure parameters $K/( {{J}_m,D} )$ leads to a failed/faster switching, where ${J}_m$ and *D* stabilize the inverse 120° triangular structure, whereas *K* introduces a small deviation to exert torques that relax the octupole to one of the six stable positions ($\varphi = \pi /6 + $ integer times of $\pi /3)$. In other words, the switching requires the torques from the current-induced spin accumulations to overcome the stable-position-favored torques due to the magnetic structure, which favors the six stable positions. Figure 4g shows that the hysteresis loop can be shifted by the *x*-direction magnetic field of ±0.0012 T (the theoretical values are usually magnitudes smaller than those in the experiments [9,13], probably because the external magnetic field is screened in the realistic polycrystals). More importantly, the simulations confirm that, without external magnetic fields and heavy metals, the all-electrical switching of the non-collinear antiferromagnet can be achieved, owing to the intrinsic non-collinear spin-orbit torques from the current-induced local spin accumulations in Mn_3_Sn itself.

## CONCLUSIONS

Our findings promise potential applications of Mn_3_Sn in information technologies. Specifically, the all-electrical control of the binary and multilevel states with large Mn_3_Sn readout signals could act as the building block for magnetic random-access memory and artificial synapses, respectively. Furthermore, in-memory computing may also be achieved by utilizing the all-electrical control of topological non-collinear antiferromagnets.

## METHODS

### Sample and device fabrication

The samples used for the current-induced switching measurements, which consist of Mn_3_Sn (50)/AlO_x_ (2) (the thicknesses shown in parentheses are in nanometers), were grown on thermally oxidized Si substrates. The top AlO_x_ layer was used as the capping layer. For comparison, reference samples consisting of Ru (3)/Mn_3_Sn (50)/AlO_x_ (2) and Ru (3)/Mn_3_Sn (50)/Pt (8)/AlO_x_ (2) were also deposited on Si/SiO_2_. The Mn_3_Sn, Ru and Pt layers were deposited at room temperature using a DC magnetron sputtering system with a base pressure of <5 × 10^–9^ Torr (at rates of ∼0.05, 0.01 and 0.02 nm/s, power of 30 W and Ar gas pressure of 0.8 mTorr). Next, the AlO_x_ layers were grown using an radio frequency (RF) magnetron sputtering system with a power of 80 W and Ar gas pressure of 2 mTorr. The Mn_3_Sn (50)/AlO_x_ (2), Ru (3)/Mn_3_Sn (50)/AlO_x_ (2) and Ru (3)/Mn_3_Sn (50)/Pt (8)/AlO_x_ (2) samples were then annealed at 450°C for 1 hour using a vacuum annealing furnace (F800-35, East Changing Technologies, China) at a base pressure of 5 × 10^–7^ Torr. The samples were patterned into Hall bar devices with a current channel width of 10 μm using standard photolithography and Ar-ion etching. For more methods of measurement and simulation, see the Methods section in the supplementary materials.

## Supplementary Material

nwac154_Supplemental_File
