# Supplementary material for: All-electrical switching of a topological non-collinear antiferromagnet at room temperature
Source: Natl Sci Rev. 2022 Aug 4;10(2):nwac154. doi: 10.1093/nsr/nwac154 (PMC9977383; doi:10.1093/nsr/nwac154)
Supplement: nwac154_Supplemental_File [file nwac154_supplemental_file.docx]

Supplementary Information for

**All-electrical switching of a topological non-collinear antiferromagnet at room temperature**

Yongcheng Deng,^1,2†^ Xionghua Liu,^1,2†^ Yiyuan Chen,^3,4†^ Zongzheng Du,^3,4†^ Nai Jiang,^1,2^ Chao Shen,^1,2^ Enze Zhang,^1,2^ Houzhi Zheng,^1,2^ Hai-Zhou Lu,^3,4*^ Kaiyou Wang^1,2,5,6*^

*^1^ State Key Laboratory for Superlattices and Microstructures, Institute of Semiconductors, Chinese Academy of Sciences, Beijing 100083, China*

*^2^ Center of Materials Science and Optoelectronics Engineering, University of Chinese Academy of Sciences, Beijing 100049, China*

^3^ *Institute for Quantum Science and Engineering and Department of Physics, Southern University of Science and Technology (SUSTech), Shenzhen 518055, China*

^4^ *Shenzhen Key Laboratory of Quantum Science and Engineering, Shenzhen 518055, China*

^5^ *Beijing Academy of Quantum Information Sciences, Beijing 100193, China*

*^6^ Center for Excellence in Topological Quantum Computation, University of Chinese Academy of Science, Beijing 100049, China*

^†^ These authors contributed equally to this work.

^*^ Correspondence and requests for materials should be addressed to H.L., or K.W.

^*^ E-mail: luhz@sustech.edu.cn, kywang@semi.ac.cn

**Table of Contents**

**Methods**

**Section S1.** X-ray diffraction results of Si/SiO_x_/Mn_3_Sn/AlO_x_

**Section S2**. Estimate of grain size

**Section S3**. Calculation of relative crystallinity

**Section S4**. Analysis of preferred orientation in polycrystalline Mn_3_Sn

**Section S5**. HR-TEM and EDX results of Si/SiO_x_/Mn_3_Sn/AlO_x_

**Section S6**. Angular dependent planar Hall Effect and positive longitudinal magnetoconductivity

**Section S7**. Temperature dependence of magnetic and transport characteristics

**Section S8.** Néel temperature of the Mn_3_Sn device

**Section S9.** Current-induced magnetization switching for reference samples

**Section S10.** Measurement of symmetry broken by nonlinear Hall effect

**Section S11**. Estimate of Joule heating

**Section S12.** Current-induced antiferromagnetic states switching without external field at temperatures of 200K and 250K

**Section S13.** Symmetry analysis on why no switching in single crystal of Mn_3_Sn with inversion symmetry

**Section S14.** Symmetry analysis to justify the Rashba spin-orbit coupling in single crystal of Mn_3_Sn with broken inversion symmetry

**Section S15.** Calculation of octupole moment from magnetic structure

**Section S16.** Effects of spin-orbit coupling, magnetic-structure parameters, and initial states on simulation results

**Section S17.** The elements of the 12×12 matrix of the s-d model Hamiltonian

**Methods**

**Microstructural characterization**. The structural analysis was carried out via X-ray diffraction using a monochromated Cu K_α_ radiation (λ = 1.54056 Å). The sample for cross-sectional scanning transmission electron microscopy characterization was prepared using a Zeiss Auriga focused ion beam system. The high-angle annular dark-field (HAADF) Z-contrast images, bright-field images, and EDX mappings were acquired using a spherical aberration-corrected FEI Titan Cubed Themis 60-300 operated at 200 kV.

**Magnetic and magnetotransport measurements.** The magnetic measurements of our polycrystalline Mn_3_Sn samples were carried out through MCD spectroscopy using a wavelength tunable laser via a supercontinuum white light source equipped with a monochromator. The reflected light from the sample was detected using a Si photodetector. The reflectance and MCD signals were obtained using two lock-in amplifiers with reference frequencies of 177 Hz and 50 kHz, respectively. Moreover, a Quantum Design vibrating sample magnetometer (VSM), Physical Property Measurement System (PPMS) with a closed-cycle helium cryostat was used to investigate the magnetic properties of our Mn_3_Sn thin film. A reading current of $I_{\mathrm{read}}=\text{0.1} \mathrm{mA}$ was used to measure the Hall resistivity $\rho_{\text{H}}$ of the Mn_3_Sn Hall bar devices, as shown in Fig. 1d. In the current-induced switching measurements, we employed a writing current pulse (with current density $J_{\text{write}}$) with a duration of 50 ms and subsequently, we applied $I_{\mathrm{read}}$ to read out the Hall voltage after waiting for 500 ms.

The angle dependence of magnetoconductivity and planar Hall effect were obtained using the horizontal rotator option of a commercial magnetoelectrical measurement system. The electrical measurements were conducted using a cryogenic probe station (Lake Shore Cryotronics, Inc). For the $\rho_{H}\text{-}\mu_{0}H_{Z}$ loop measurements shown in Fig. 2e, we first initialized the antiferromagnetic states by applying a saturation field of 1.8 T, and we then scanned $\mu_{0}H_{Z}$ from 1.8 T to $\mu_{0}H_{Z}^{\min}$ and back to 1.8 T. Similarly, for the $\rho_{H}\text{-}J_{\text{write}}$ loop measurements shown in Fig. 2f, we first initialized the antiferromagnetic states using a positive current pulse (with a current density of 6 × 10^6^ A∙cm^-2^ larger than the switching current density $J_{c}$). We then scanned $J_{\text{write}}$ from 6 × 10^6^ A∙cm^-2^ to $J_{\text{write}}^{\min}$ and back to 6 × 10^6^ A∙cm^-2^ at zero magnetic field.

For the nonlinear Hall measurements, a sinusoidal current with a frequency of 133.33 Hz along the longitudinal direction was applied to the Hall devices using standard lock-in techniques (Stanford Research Systems Model SR830), the second harmonic voltages were detected in a direction perpendicular to the current. All nonlinear Hall measurements were carried out at room temperature without external magnetic field. The relationship between the nonlinear Hall voltage and current was obtained by gradually sweeping the amplitude of the sinusoidal current.

**Simulating current-induced switching of the non-collinear antiferromagnet.**

To understand the microscopic mechanism of the all-electrical switching of the non-collinear antiferromagnet, we performed a numerical simulation of the octupole polarization direction $\boldsymbol{O=(}O_{x}\boldsymbol{,}O_{z}\boldsymbol{)}$, which can be determined from the direction of the magnetic moment $\mathbf{m}_{i2}$ (assumed to be the same for different unit cells $i$ in the simulation) of the second Mn atom in the unit cell as

$O_{x}=\mathbf{(m}_{i2})_{x}\boldsymbol{,}O_{z}=\mathbf{-(m}_{i2})_{z}$,

where $i$ indexes the unit cells. This expression is simplified from the full definition in Supplementary Section S15. The simulation procedures are as follows.

**Step 1** Assume an initial magnetic structure of the Mn magnetic moments $\mathbf{m}_{ia}$ (Eq. 1 in Methods), $a=\{1, 2, 3\}$ denotes the three Mn atoms in each unit cell.

**Step 2** Use the real-space form of an s-d model (Eq. 2 in Methods) to describe the itinerant electrons in the local Zeeman fields exerted by the Mn magnetic moments $\mathbf{m}_{ia}$, where s for the itinerant electrons and d for the Mn magnetic moments.

**Step 3** For an input electric current $I_{x}$, use the linear-response theory for the current-spin correlation (Eqs. 3 and 4 in Methods) to calculate the local spin accumulations of the itinerant electrons induced by the current $I_{x}$.

**Step 4** Use the local spin accumulations $\mathbf{S}_{a}$of the itinerant electrons to calculate the magnetic torques $\mathbf{T}_{ia}$ exerted on the Mn magnetic moments $\mathbf{m}_{ia}$ (Eq. 5 in Methods).

**Step 5** Use the magnetic torques $\mathbf{T}_{ia}$ and the Landau-Lifshitz-Gilbert equation (Eq. 6 in Methods) to calculate a new magnetic structure of $\mathbf{m}_{ia}$.

**Step 6** Return to Step 1 with the new magnetic structure.

Repeat steps 1-6 until the magnetic structure converges to yield the octupole moment in the presence of the injected current $I_{x}$. Any crystal grain configuration can be constructed using the three vector grain configurations of our polycrystalline Mn_3_Sn Hall-bar devices (Fig. 4a). Here we focus on the *z-x* configuration (left-hand side in Fig. 4a) because we found that the switching accompanied by the reversing of the anomalous Hall resistivity due to the electric current occurs only in this configuration.

**Magnetic structure of Mn moments.** To understand the microscopic mechanism of the current-induced switching of the non-collinear antiferromagnetic states, we study the dynamics of the sublattice moments $\mathbf{m}_{ia}$ using the following Hamiltonian defined on a single layer of the Mn_3_Sn kagome lattice [1](on the $x\text{-}z$ plane in Fig. 4a)

$H_{m}=D\sum_{\left\langle i,j \right\rangle} \mathbf{c}\cdot(\mathbf{m}_{i1}\times\mathbf{m}_{j2}+\mathbf{m}_{i2}\times\mathbf{m}_{j3}+\mathbf{m}_{i3}\times\mathbf{m}_{j1})+J_{m}\sum_{\left\langle ia,jb \right\rangle} \mathbf{m}_{ia}\cdot\mathbf{m}_{jb} -K\sum_{ia} (\mathbf{k}_{a}\cdot\mathbf{m}_{ia})^{2}$, (1)

where $i$ and $j$ index the unit cells, $a, b= \{1, 2, 3\}$ denote the sublattices, and $\mathbf{c} = (0, 1, 0)$is the unit vector along the y axis (Fig. 4a). This model contains only three Mn atoms in a unit cell, and we neglect inter-layer couplings because they do not provide qualitatively new effects. The Dzyaloshinskii-Moriya interaction $D$, nearest neighbour exchange interaction $J_{m}$, and in-plane magnetic anisotropy $K$ are assumed to be positive, and they stabilize the inverse triangular spin texture of Mn_3_Sn. $\mathbf{m}_{ia}$ represents a unit magnetic moment on site $ia$. $\mathbf{k}_{a}=\left( \sin\phi_{a},0,\cos\phi_{a} \right)$, with $\left( \phi_{1},\phi_{2},\phi_{3} \right)=\frac{(\pi,9\pi,5\pi)}{6}$. The *K* term lifts the in-plane U(1) degeneracy and fixes the sixfold symmetry, introducing six stable positions for the octupole. For the magnetic structure, we used a single unit cell with periodic boundary conditions, the same as that in the previous work [1].

**s-d model**. To establish the relationship between the injected current and the local Mn magnetic moments and calculate the current-induced local spin accumulations, we employed an s-d model from the previous work [2]

$$H_{sd}=t\sum_{\left\langle ia,jb \right\rangle n} c_{ian}^{\dagger}c_{jbn}+J_{sd}\sum_{iann^{'}} \mathbf{m}_{ia}\cdot\boldsymbol{\sigma}_{nn^{'}}c_{ian}^{\dagger}c_{ian^{'}}+\Lambda\sum_{in,a=\{1,2,3\}} c_{ian}^{\dagger}c_{ian}$$

$+\lambda_{R}\sum_{\left\langle ia,jb \right\rangle nn^{'}} i\left( \overset{\wedge}{\boldsymbol{y}}\times{\overset{\wedge}{\boldsymbol{d}}}_{ia,jb} \right)\cdot\boldsymbol{\sigma}_{nn^{'}}c_{ian}^{\dagger}c_{jbn^{'}}$, (2)

where $c_{ian}$ is the annihilation operator of the electron highly localized on site *ia* (notice that sublattice index $a=\{1,2,3,1^{'},2^{'},3^{'}\}$ here corresponds to a monolayer AB stack Mn_3_Sn structure and we only need to calculate the local spin accumulations on sublattice 1,2,3 for the simulation) with spin $n=\left\{ \uparrow,\downarrow\right\}$, $t$ is the kinetic energy, $\Lambda$ is the energy difference between the $\left\{ 1,2,3 \right\}$ and $\{1^{'},2^{'},3^{'}\}$ layers, $J_{sd}$ is the exchange interaction between the local moments and the spins of itinerant electrons, $\boldsymbol{\sigma}$is the spin operator of the electrons, ${\overset{\wedge}{\boldsymbol{d}}}_{ia,jb}$ is the unit vector from site $ia$ to $jb$, and $\lambda_{R}$ measures the Rashba-type spin-orbit coupling that breaks the mirror time reversal symmetry and inversion symmetry. By performing a Fourier transformation, the Hamiltonian in Eq. 2 can be converted from the real space to the *k* space. For each *k* point in the *k* space, the Hamiltonian is a *k* labeled 12 × 12 matrix (See Supplementary Section S17). Diagonalizing the matrix provides us with the energy spectrum and eigenstates of the s-d model. In Supplementary Section S14, we show that a reasonable energy difference ( $\Lambda=\text{0.1}t$, comparable with the Rashba spin-orbit coupling) does not cause qualitative change to our switching mechanism. To simplify the illustration of the switching mechanism, we assume $\Lambda=\text{0}$ in the simulations shown in our main text.

**Current-induced spin accumulations and torques**. Using the linear-response theory, the Kubo formula for the local spin accumulations on sublattice $a$ in response to the electric field (i.e., the injected write current) along the $\beta=\left\{ x,y,z \right\}$ direction can be written as

$\delta\left\langle\mathbf{s}_{a} \right\rangle_{d}=-\frac{e\hbar}{2}\tau E^{\beta}\sum_{n} \int\frac{d\mathbf{k}}{(2\pi)^{2}}\frac{\partial f}{\partial\varepsilon_{n}}\upsilon_{nn}^{\beta}\boldsymbol{\sigma}_{nn}^{a},$ (3)

$\delta\left\langle\mathbf{s}_{a} \right\rangle_{\mathrm{od}}=-\frac{e\hbar^{2}}{2}E^{\beta}\sum_{m\neq n} \int\frac{d\mathbf{k}}{(2\pi)^{2}}\left( f_{m}-f_{n} \right)\times\mathrm{Im} \left[ \frac{\upsilon_{mn}^{\beta}\boldsymbol{\sigma}_{nm}^{a}}{(\varepsilon_{m}-\varepsilon_{n})^{2}} \right],$ (4)

where “d” stands for diagonal, and “od” stands for off-diagonal. The electric field can be derived from the injected current $I$ as $E=\frac{m^{*}I}{\omega dn_{3D}e^{2}\tau}$, where $m^{*}$ is the electron effective mass, $\omega$ and $d$ are the width and thickness of the device, respectively, $n_{3D}$ is the carrier density, $e$ is the elementary charge, $\tau$ is the relaxation time, $f$ is the Fermi-Dirac distribution function, $\varepsilon$ is the energy of state, $\upsilon$denotes the velocity operator, and $\boldsymbol{\sigma}^{a}=\left( \sigma_{x}^{a},\sigma_{y}^{a},\sigma_{z}^{a} \right)$ represents the local spin operator on sublattice *a*. In Fig. 4c, the unit of the spin accumulation is $s_{0}=I_{x} m^{*}/2 n_{3D} e a_{L}$, with $a_{L}$ being the kagome plane lattice constant. In the calculation of the local spin accumulations, we used 50 × 50 unit cells, which is sufficient to yield convergent results. The local spin accumulations on the sublattice produce a field-like torque [3]

$\mathbf{T}_{ia}=-\left| \gamma\right|\mathbf{m}_{ia}\times\mathbf{H}_{a}^{I},$ with $\mathbf{H}_{a}^{I}=-\frac{J_{\mathrm{sd}}}{M_{S}}\frac{\mathbf{S}_{a}}{\frac{\hbar}{2}},$ (5)

where $\mathbf{S}_{a}=A_{U}\times\delta\left\langle\mathbf{s}_{a} \right\rangle_{d}$ is the induced local spin accumulations on the sublattice $a$ of a unit cell with area $A_{U}$, and $M_{S}=3\mu_{B}$ is the saturation magnetic moment of an Mn atom. $\delta\left\langle\mathbf{s}_{a} \right\rangle_{\mathrm{od}}$ are not included because in the presence of the Rashba spin-orbit coupling, the spin accumulation calculated from Eq. 4 on an Mn atom is always opposite to that of its diagonal counterpart in the other kagome layer (see the symmetry analysis in Supplementary Section S14). In other words, the off-diagonal spin accumulations do not contribute to the switching.

**Landau-Lifshitz-Gilbert (LLG) equation**. The LLG equation describing the dynamics of the magnetic moments on the sublattices of a single layer of the kagome lattice in Mn_3_Sn is

${\dot{\mathbf{m}}}_{ia}=-\left| \gamma\right|\mathbf{m}_{ia}\times\mathbf{H}_{\mathrm{eff},ia}+\alpha\mathbf{m}_{ia}\times{\dot{\mathbf{m}}}_{ia}+\mathbf{T}_{ia}\boldsymbol{,}$ (6)

where the suffix $i$ represents a unit cell, and $a = \{1, 2, 3\}$ indexes a sublattice. The effective magnetic field is given by $\mathbf{H}_{\text{eff,}ia}=-M_{S}^{-1}\delta H_{m}/\delta\mathbf{m}_{ia}$, with $H_{m}$ being the Hamiltonian of the magnetic structure (Eq. 1). The first and second terms in the right-hand side of Eq. 6 represent the gyroscopic torque and Gilbert damping torque, respectively, $\gamma(\gamma<0)$ is the electron gyromagnetic ratio, and $\alpha$ is the Gilbert damping coefficient. The third term $\mathbf{T}_{ia}$ is the spin torque (Eq. 5) acting on the moments of the corresponding sublattices. Using this equation, we can simulate the dynamics of the magnetic moments on the sublattices to understand how *J*_write_ influences the polarization of the octupole and determines the sign of the anomalous Hall resistivity, as shown in Fig. 4b. In Fig. S16 we show that our switching mechanism is not influenced by initial state of magnetic structure, i.e., the final state of the switching is determined by the direction of injected current. To numerically solve the LLG equation, we used ODE23 Matlab function, in which the time scale is self-adapted, i.e., it changes depending on the precision requirement.

**Simulation parameters.** In Fig.4, unless specified on the curves, the simulation parameters are $t= \text{0.25} \text{eV}$, $J_{sd}=\text{0.375} \text{eV}$, $\Lambda=\text{0}$, $\lambda_{R}=\text{0.2} \text{t}$, $\tau=\hbar/\text{2}\Gamma$ with $\Gamma=\text{1.25} \text{meV}$, $m^{*}=\text{4.05}\text{ }\text{×}\text{ }\text{1}\text{0}^{\text{-31}}\text{kg}$, $\omega=\text{15} \text{μm}$, $d=\text{40} \text{nm}$, $n_{\text{3}D}=\text{6 × 1}\text{0}^{\text{23}}\text{cm}^{\text{-3}}$, $T=\text{290 K}$, $J_{m}=\text{23 meV}$, $D=\text{1.6 meV}$, $K=\text{0.17 meV}$, $\alpha=\text{0.003}$ [1,4]. Using these parameters in the simulations, a critical switching current of $I=\text{±30 mA}$ is obtained, which corresponds to the current density $J_{\mathrm{write}} \text{≈}\text{ }\text{5}\text{ }\text{×}\text{ }\text{1}\text{0}^{\text{6}}\text{A}\text{∙}\text{c}\text{m}^{\text{-2}}$ in the measurement. The $K/{(J}_{m}, D)$ values used in Fig. 4f are 0.085/(23,1.6), 0.17/(46,1.6), and 0.17/(23,3.2) for the above-critical curves and 0.34/(23, 1.6), 0.17/(11.5, 1.6), and 0.17/(23,0.8) for the below-critical curves, all in units of meV.

**Section S1. X-ray diffraction results of Si/SiO_x_/Mn_3_Sn/AlO_x_**

The crystallinity of Si/SiO_x_/Mn_3_Sn (100 nm)/AlO_x_ film is examined by the XRD method. Obviously, we can see most diffraction peaks of Mn_3_Sn, the two strongest peaks (002), (021) at 40°and 42°, as well as (010) and (020) at 18° and 36°, which verify the existence of stable D0_19_ phase in our films.


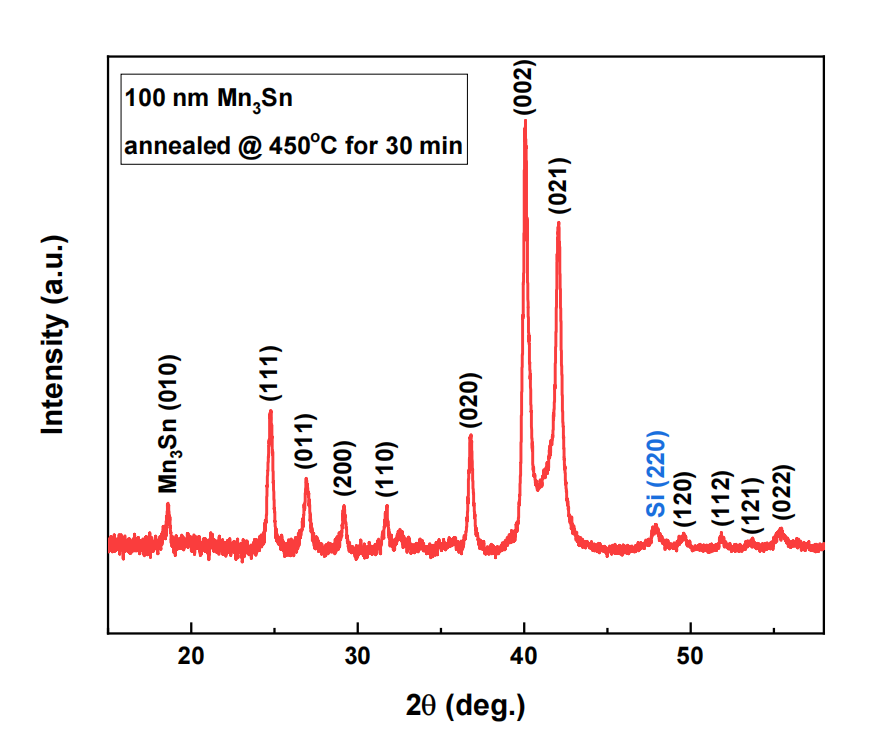
**Supplementary Fig. S1 | The X-ray diffraction measurement for the 100-nm-thick film of Mn_3_Sn on a Si/SiO_2_ substrate.** All the peaks can be indexed by the hexagonal D0_19_ Mn_3_Sn structure and no additional peaks coming from plausible impurity phases.

**Section S2. Estimate of grain size**

We estimate the grain size in our Mn_3_Sn thin films by using the Scherrer equation to analyze the XRD data in Supplementary Fig. S1. The Scherrer equation [5] is given as

$D=\frac{K\lambda}{\beta\cos\theta}$, (S1)

where *D* is grain size (unit is nm), *K* is Scherrer constant, which is equal to 0.9, $\lambda$ is wavelength of the x-ray sources, $\lambda$= 0.15406 nm, $\beta$ is the full width at half maximum (FWHM, in units of rad) of the diffraction peak, *θ* is the peak position in rad.

Take the logarithm of both sides of Eq. S1,

$\ln\left( \beta\right)=\ln\left( \frac{1}{\cos\theta} \right)+\ln\left( \frac{K\lambda}{D} \right)$. (S2)

We can fit the positions and FWHM of the three main peaks by using Eq. S2, as shown in Table S1 below.

**Table S1.** Peak positions and FWHM of the three main peaks fitted from the XRD data in Supplementary Fig. 1 by using the Scherrer equation.

| peak position (deg.) | FWHM (deg.) |
| --- | --- |
| 36.79 | 0.25 |
| 40.10 | 0.46 |
| 42.04 | 0.73 |

By linear fitting Table S1, we obtain an intercept of $\text{-4.56 ± 0.17}$ and the grain size of $\text{13.23 ± 2.48}$ nm, which is smaller than the thickness of the film.

**Section S3. Calculation of relative crystallinity**

To quantitatively characterize the quality of our samples, we calculate the relative crystallinity, which can be defined as [6]

$X_{c}=\frac{W_{c}}{W_{c}+W_{a}}$, (S3)

where the weight of the crystalline phases $W_{c}$ is proportional to the area of crystalline peaks and the weight of the amorphous phase $W_{a}$ is proportional to the area of the rest part of the XRD data in Supplementary Fig. S1. There are mainly three strong diffraction peaks (021), (002), and (020) in our samples, so we choose an angle range of 36 - 50° for the calculation. Please note that the crystallinity calculated here is relative, because there are also other peaks in our XRD patterns, such as those from Si and other amorphous phases.

**Table S2.** Area of the peaks and relative crystallinity from the XRD data in Supplementary Fig. S1.

| Area of (020) | Area of (002) | Area of (021) | All area | ratio |
| --- | --- | --- | --- | --- |
| 276.35 | 1235.63 | 1426.13 | 3106.94 | 94.57% |

According to Table S2, peak (021) is the strongest, peak (002) is the second, and peak (002) is the weakest. The calculated crystallinity is 94.57%, which verifies the high quality of our polycrystalline films.

**Section S4. Analysis of preferred orientation in polycrystalline Mn_3_Sn**

The diffraction of the polycrystal can be regarded as the summation of those of three basic-vector single crystals [7]. For the hexagonal Mn_3_Sn, basic-vector [020] and [200] have the same symmetry, so we simulate single-crystal diffraction in three resultant vector directions, **a** [020], **b** [002], **c** [021]. The three resultant vectors are not collinear in pairs. The observed diffraction intensity of peak $i$ can be expressed as

$D^{i}=\sum_{j=a,b,c} D_{j}^{i}\cdot P_{j}$, (S4)

where $i$ stands for the three main peaks (020), (002), and (021) in our samples, $j$ represents three basic-vector directions (**a** [020], **b** [002], and **c** [021]), $D_{j}^{i}$ is the simulated intensity of peak $i$ along the $j$ direction, and $P_{j}$ represents the weights of three resultant vector single crystals. Eq. S4 can be explicitly expressed as

$\left\{ \begin{matrix} D^{(020)}=D_{a}^{(020)}\cdot P_{a}+D_{b}^{\left( 020 \right)}\cdot P_{b}+D_{c}^{\left( 020 \right)}\cdot P_{c}, \\ D^{(002)}=D_{a}^{(002)}\cdot P_{a}+D_{b}^{\left( 002 \right)}\cdot P_{b}+D_{c}^{\left( 002 \right)}\cdot P_{c}, \\ D^{(021)}=D_{a}^{(021)}\cdot P_{a}+D_{b}^{\left( 021 \right)}\cdot P_{b}+D_{c}^{(021)}\cdot P_{c}. \end{matrix} \right.$ (S5)

Then we simulate the XRD pattern of the three resultant vector single crystals by assuming a preferred orientation and alignment. In the simulation, the x-ray wavelength is 1.541 Å, Pseudo-Voight function is used to generate the diffraction peak. In Supplementary Fig. S2, the diffraction of the single crystal varies greatly with the preferred orientation. The alignment is measured by a number between 0 and 1, with 0 (1) representing a fully random (ordered) sample. In Supplementary Fig. S2, as the alignment decreases, the diffraction peaks (except (020), (002) and (021)) gradually increase, which means the larger alignment the stronger the main peak and the smaller the miscellaneous peaks. From our experimental results shown in Supplementary Fig. S1, in the range of 30 - 50°, there are almost no diffraction peaks except (020), (002), and (021), so we chose a large alignment, such as 0.8 and 0.9. The calculated diffraction intensity $D_{j}^{i}$ of each peak is shown in Supplementary Fig. S2. The area of (020), (002) and (021) integrated from Supplementary Fig. S1 are 276.35, 1235.63, and 1426.13, respectively.

For the alignment = 0.9, put the area of the (020), (002) and (021) peaks into Eq. S5, we obtain

$$\left\{ \begin{matrix} 276.35=939.485\cdot P_{a}+0.089\cdot P_{b}+0.355\cdot P_{c}, \\ 1235.63=0.093\cdot P_{a}+2949.704\cdot P_{b}+0.137\cdot P_{c}, \\ 1426.13=1.442\cdot P_{a}+0.532\cdot P_{b}+1907.237\cdot P_{c}. \end{matrix} \right.$$

The ratio of the in-plane to out-of-plane case is about ($P_{a}+P_{c}$)/$P_{b}$ = 2.49.

For the alignment = 0.8,

$$\left\{ \begin{matrix} 276.35=329.22\cdot P_{a}+0.705\cdot P_{b}+2.734\cdot P_{c}, \\ 1235.63=0.738\cdot P_{a}+1031.49\cdot P_{b}+1.082\cdot P_{c}, \\ 1426.13=11.098\cdot P_{a}+4.198\cdot P_{b}+670.54\cdot P_{c}. \end{matrix} \right.$$

The ratio of the in-plane to out-of-plane case is about ($P_{a}+P_{c}$)/$P_{b}$ = 2.44, similar to that of the alignment = 0.9.

According the XRD analysis, we find that our Mn_3_Sn thin films have the in-plane preferred alignment orientation.


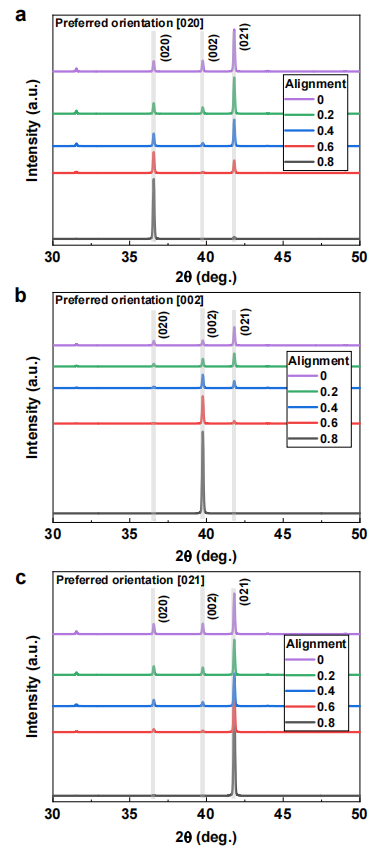


**Supplementary Fig. S2 | Simulated x-ray diffraction patterns with alignment for preferred orientations.** XRD results for preferred orientation [020] (**a**), [002] (**b**), and [021] (**c**), respectively, for alignment 0, 0.2, 0.4, 0.6, and 0.8. The corresponding peaks vary with the alignment for different preferred orientations.

**Section S5**. **HR-TEM and EDX results of Si/SiOx/Mn_3_Sn/AlO_x_**

The microstructural characterization was carried out by cross-sectional HR-TEM. As showed in Fig S3(a), there is a clear boundary between the continuous Mn_3_Sn film and amorphous SiO_x_ substrate, suggesting the textured polycrystalline Mn_3_Sn can be obtained by post-annealing without a buffer layer. The spatial distribution of the intensity for Si, Mn, Sn, and Al elements are represented by different colors in Fig. S3(b). Mn and Sn are detected at the same sample regions, therefore confirming the homogeneity of the Mn_3_Sn film, and the chemical composition of Mn_3.06_Sn_0.94_ is revealed by the EDX result. It has been reported that stable D0_19_ phase can exist in the stoichiometric ratio of Mn : Sn in the range of 3.02 : 0.98 to 3.15 : 0.85 [8], which verified the purity of our Mn_3_Sn films together with the XRD results.


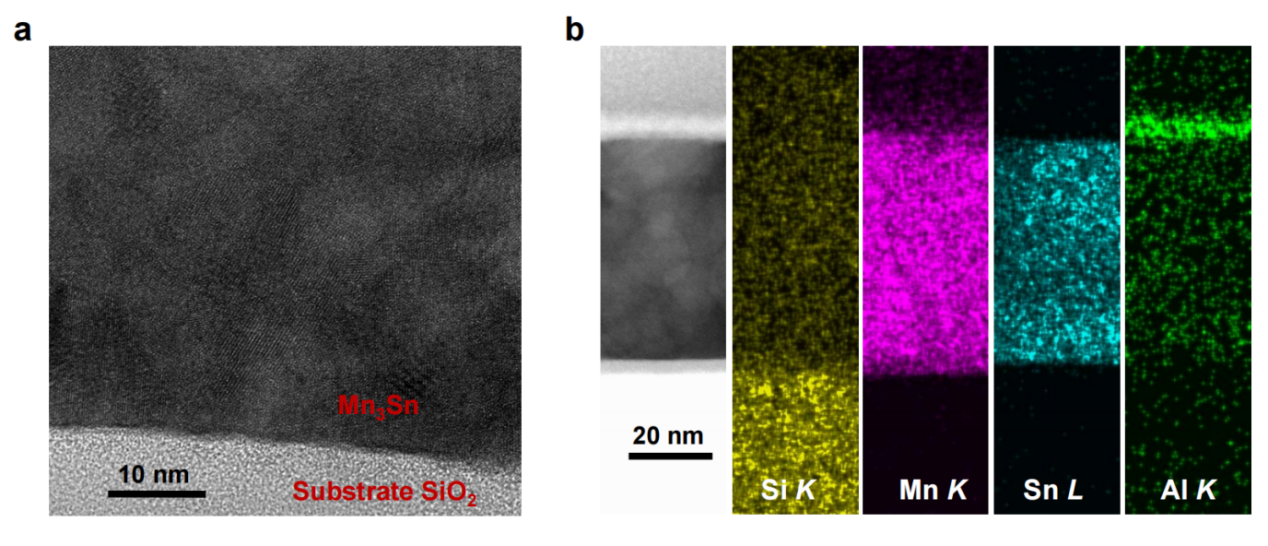


**Supplementary Fig. S3 | Microstructural characterization of the 50-nm-thick Mn_3_Sn thin on a Si/SiO_2_ substrate.** (a) Cross-sectional HR-TEM image of the 50 nm Mn_3_Sn film on Si/SiO_2_. (b) Cross-sectional HAADF-STEM images (left) and the corresponding EDX maps (right). The chemical composition of Mn_3.06_Sn_0.94_ is obtained for our thin film.

**Section S6. Angular dependent planar Hall Effect and positive longitudinal magnetoconductivity**

It is believed that the violation of a conservation law of left- and right-handed 3D Weyl fermions can result in the angular dependent planar Hall effect [9] and positive longitudinal magnetoconductivity [10]. The angular dependent magnetoconductivity is related to the scalar product ($\left| \mathbf{E}\cdot\mu_{0}\mathbf{H} \right|$) of the magnetic field $\mu_{0}\mathbf{H}$ and electric field $\mathbf{E}$. The in-plane magnetoconductivity *σ* and planar Hall conductivity $\sigma_{H}^{\text{PHE}}$ are expressed as

$\sigma=\sigma_{\perp}+\Delta\sigma_{\text{Chiral}}\cos^{2} \Phi$, (S6)

$\sigma_{H}^{\text{PHE}}=\Delta\sigma_{\text{Chiral}}\sin\Phi\cos\Phi$. (S7)

Here $\Delta\sigma=\sigma-\sigma_{\perp}=\Delta\sigma_{\text{Chiral}}\cos^{2} \Phi$ is the angular dependent magnetoconductivity resulted from the chiral anomaly of Weyl fermions, $\sigma_{\perp}$ is the magnetoconductivity when the electrical current is normal to ($\Phi=90^{o}$) the magnetic field in the plane, $\Delta\sigma_{\text{Chiral}}$ is the maximum change of the magnetoconductivity due to the chiral anomaly, and $\Phi$ is the angle between the magnetic field and electrical current in the plane, as presented in the inset of Fig. 1c. We find that the angular dependence of the magnetoconductivity and planar Hall conductivity with a fixed magnetic field of 1.8 Tesla rotated in the plane are consistent with the theory of chiral anomaly and well fitted by Eqs. S6-S7, and the same $\left| \Delta\sigma_{\text{Chiral}} \right|$ value of around 0.32 $\Omega^{-1}\text{c}\text{m}^{-1}$ is obtained for both $\Delta\sigma\left( \Phi\right)$ and $\sigma_{H}^{\text{PHE}}\left( \Phi\right)$.

In addition, the angular dependence of the magnetoconductivity with the magnetic field applied in the $x\text{-}z$ plane is also in good agreement with the chiral anomaly of Weyl fermions, as exhibited in Supplementary Fig. S4. The longitudinal magnetoconductivity for a magnetic field parallel to the current ($\mu_{0}\mathbf{H || E}$) increases with $|\mu_{0}\mathbf{H}|$. On the other hand, a magnetic field perpendicular to the plane of the device ($\mu_{0}\mathbf{H}\perp\mathbf{E}$, $\Theta=90^{o}$) produces a smaller negative magnetoconductivity.


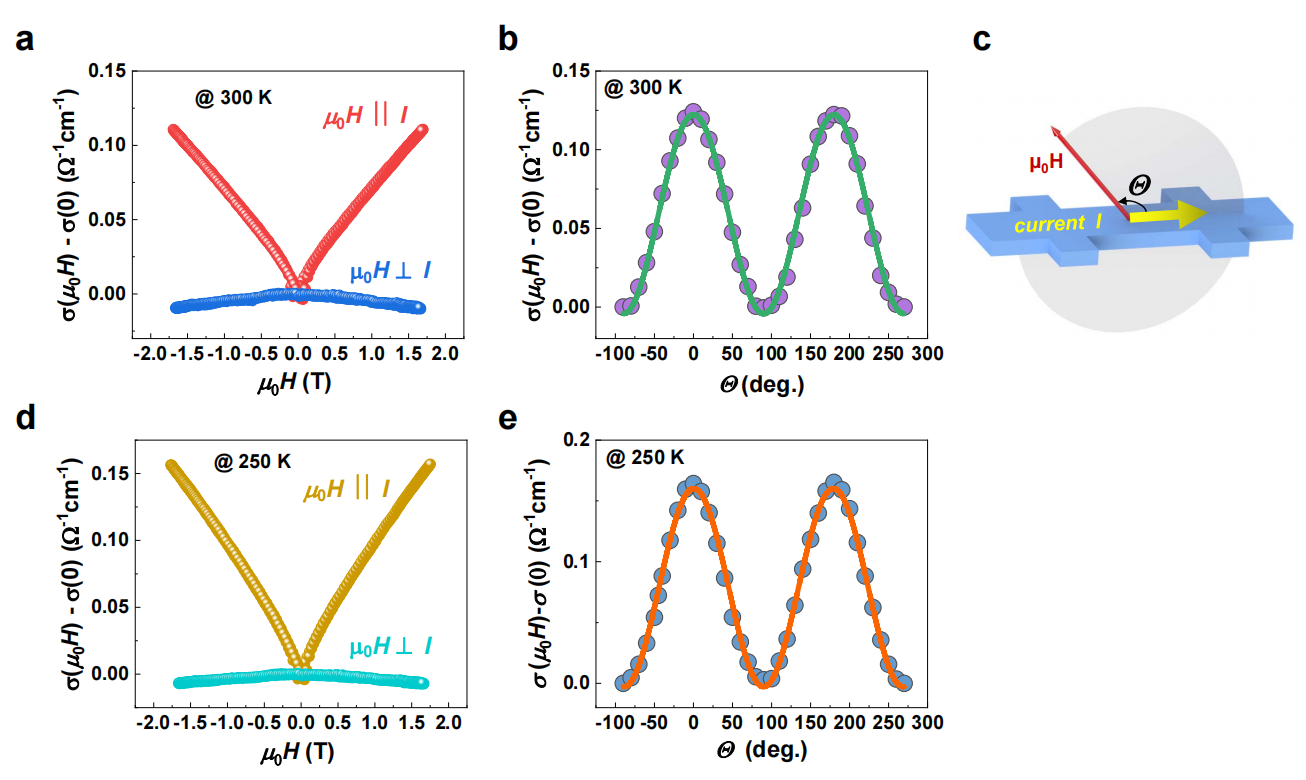


**Supplementary Fig. S4 | Angular dependent longitudinal magnetoconductivity measurements for the 50-nm-thick Mn_3_Sn device in the** $\boldsymbol{x}\mathbf{-}\boldsymbol{z}$ **plane.** (a), (d) The magnetoconductivity $(\sigma\left( \mu_{0}H \right)-\sigma\left( 0 \right))$ for the 50-nm-thick Mn_3_Sn device as a function of the magnetic field $\mu_{0}H$ parallel or perpendicular to the current $I$at 300 K (a) and 250 K (d), respectively. Here $\sigma\left( 0 \right)$is the magnetoconductivity at 0 Tesla. (b), (e) Angular $(\Theta)$ dependence of the magnetoconductivity $(\sigma\left( \mu_{0}H \right)-\sigma\left( 0 \right))$ at 300 K (b) and 250 K (e), respectively. (c) The schematic experimental setup for the magnetotransport measurements.

**Section S7.** **Temperature dependence of magnetic and transport characteristics**


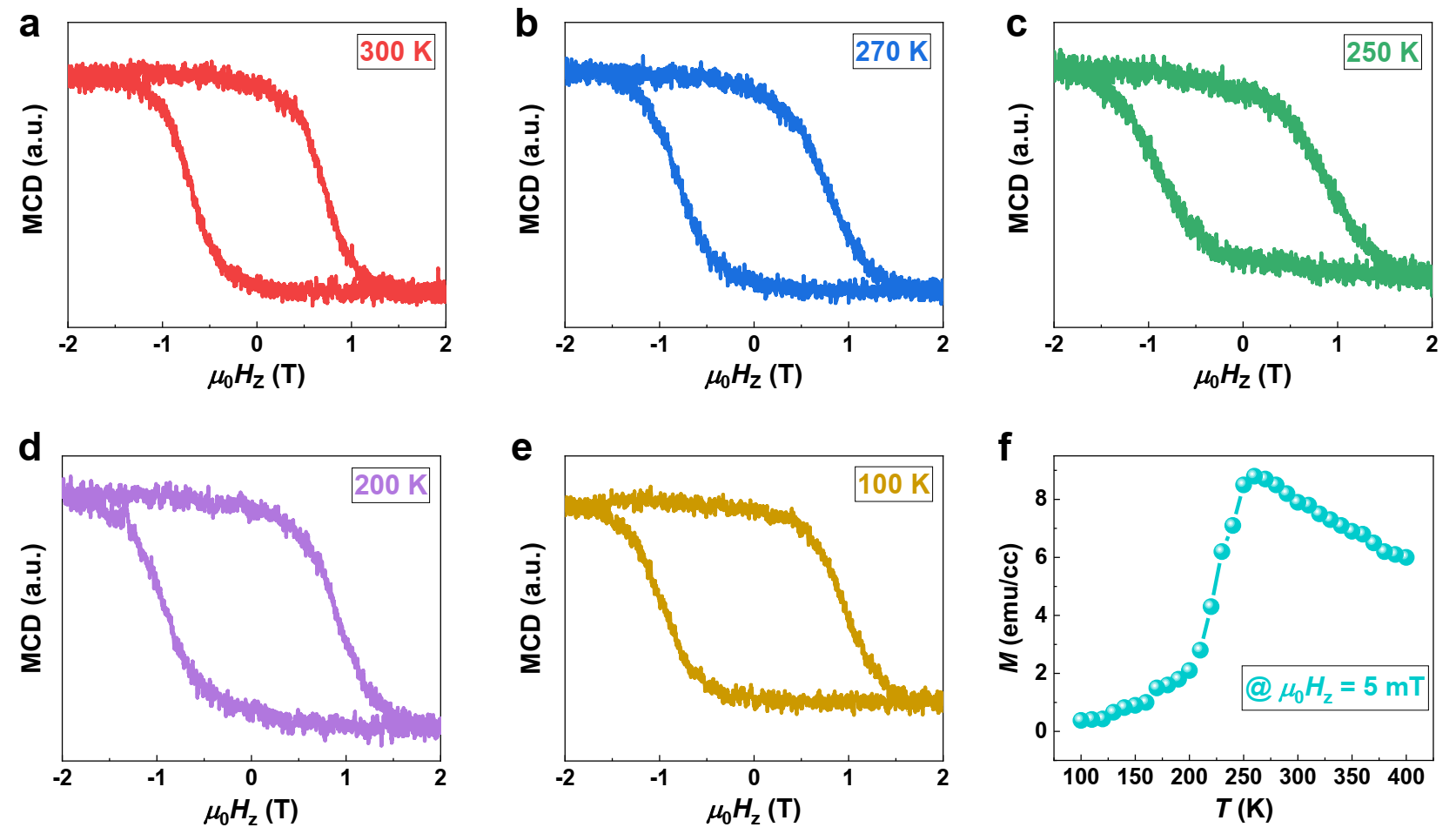


**Supplementary Fig. S5 | Effect of temperature on magnetism for the 50-nm-thick Mn_3_Sn sample.** Magnetic circular dichroism signal versus $\mu_{0}H_{Z}$ at 300 K (**a**), 270 K (**b**), and 250 K (**c**), 200 K (**d**), 100 K (**e**), respectively. The clear hysteresis loops are observed at different temperatures. The coercive field gradually increases with decreasing temperature. (**f**) The measured magnetization versus temperature at $\mu_{0}H_{z}=\text{5}\mathrm{mT}$ using a vibrating sample magnetometer. A rapid decrease in magnetization around 250 K would correspond to the transition to the spiral phase.


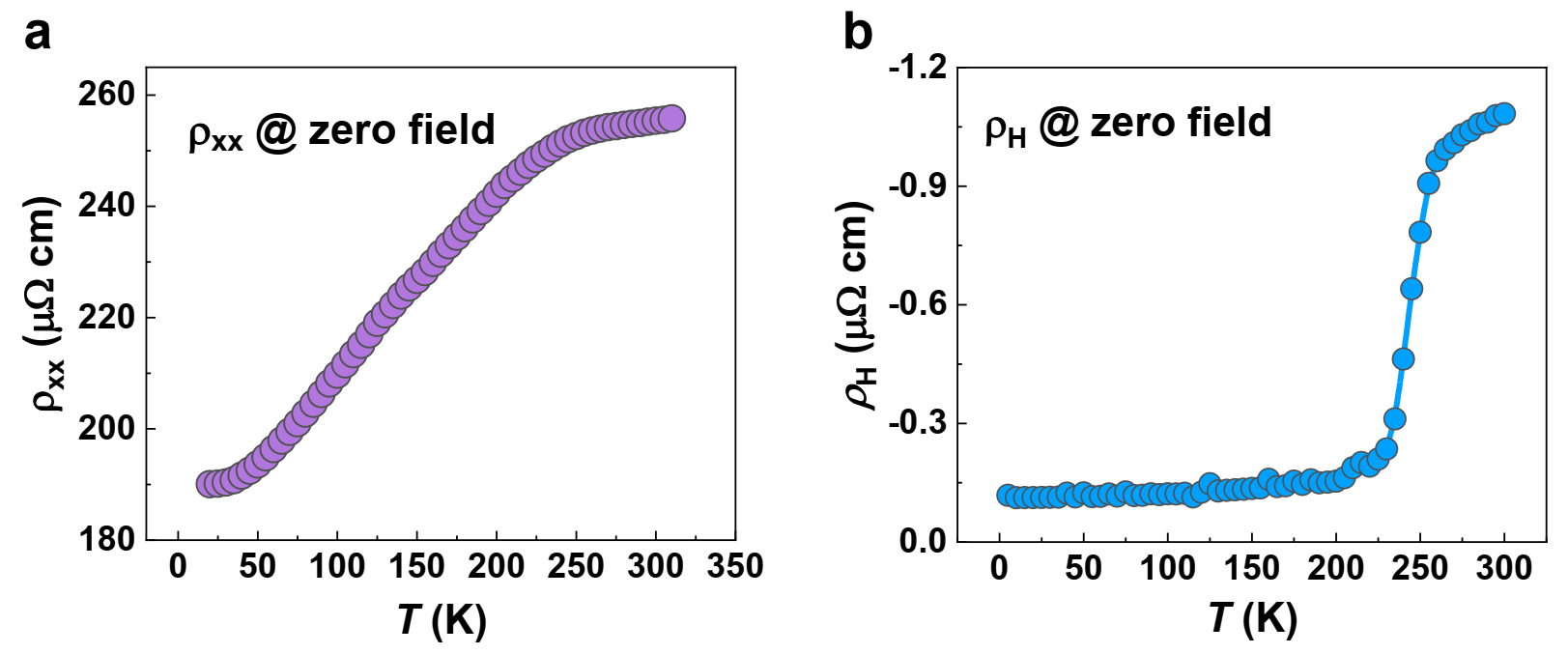


**Supplementary Fig. S6 | Effect of temperature on transport for the 50-nm-thick Mn_3_Sn sample.** Longitudinal resistivity $\rho_{xx}$ (a) and Hall resistivity $\rho_{H}$ at zero magnetic field (b) as a function of temperature. The behavior of $\rho_{xx} (T)$ and $\rho_{H}(T)$ are similar to those in the previous works [8,11].

**Section S8. Néel temperature of the Mn_3_Sn device**

**

**

**Supplementary Fig. S7 | Anomalous Hall resistivity as a function of** $\boldsymbol{\mu}_{\boldsymbol{0}}\boldsymbol{H}_{\boldsymbol{Z}}$ **for the 50-nm-thick Mn_3_Sn device at different temperatures ranging from 330 to 435 K.** Both $\Delta\rho_{H}^{0}$ and $\mu_{0}H_{C}$ gradually decrease with increasing temperature and become nearly zero at 430 K, which corresponds to the Néel temperature of the Mn_3_Sn device. The temperature dependence of $\Delta\rho_{H}^{0}$ at zero field and $\mu_{0}H_{C}$ from the AHE hysteresis loops are summarized in Fig. 1f.

**Section S9. Current-induced magnetization switching for reference samples**

We also investigated the current-induced deterministic switching of the reference samples. The observations are consistent with the literature [1]. At zero magnetic field, no current-induced deterministic switching was observed for both the Ru/Mn_3_Sn and Ru/Mn_3_Sn/Pt samples. Under the external magnetic field of ${\mu_{0}H}_{x}$ = ±0.2 T, there is still no deterministic switching was observed for the Ru/Mn_3_Sn sample. However, the current-induced deterministic magnetization switching was observed for the Ru/Mn_3_Sn/Pt sample under ${\mu_{0}H}_{x}$ = ±0.1 T, where the switching direction is opposite for opposite external magnetic fields. The results indicate the dominance of the spin Hall effect in the current-induced switching of the Ru/Mn_3_Sn/Pt device.


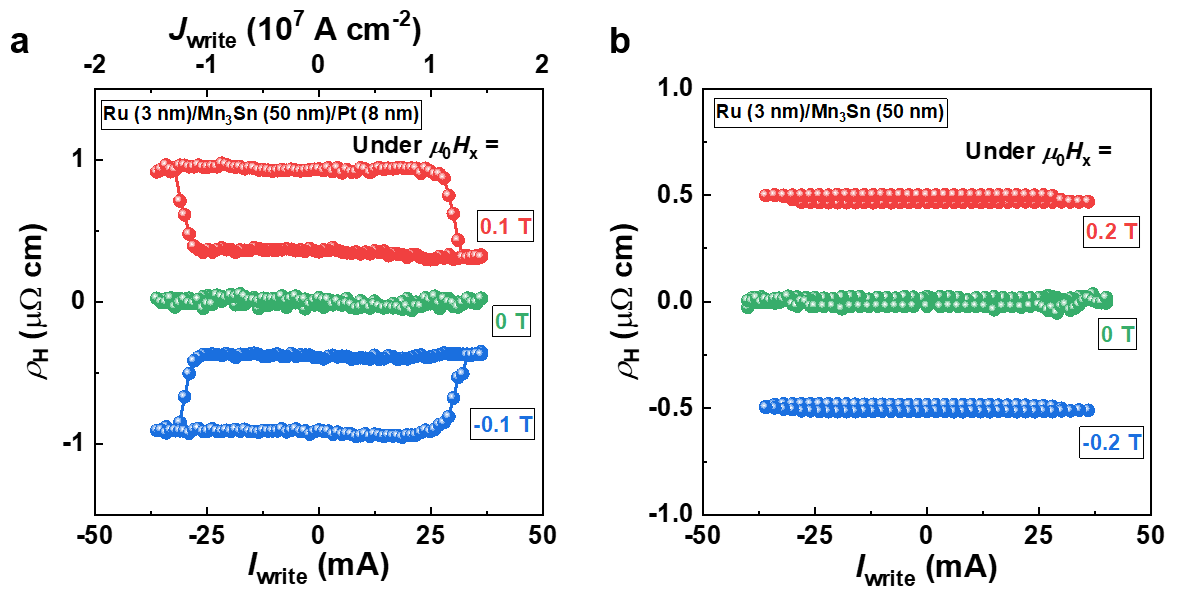


**Supplementary Fig. S8 | Current-induced antiferromagnetic states switching in the Ru/Mn_3_Sn/Pt and Ru/Mn_3_Sn devices in an external magnetic field** ${\boldsymbol{\mu}_{\boldsymbol{0}}\boldsymbol{H}}_{\boldsymbol{x}}$**.** (a) Anomalous Hall resistivity $\rho_{H}$ as a function of the writing current (with density of $J_{\mathrm{write}}$) in the Ru (3 nm)/Mn_3_Sn (50 nm)/Pt (8 nm) device at 0.1 T (red), -0.1 T (blue) and zero field (green). (b) $\rho_{H}$ vs $J_{\mathrm{write}}$ in the Ru (3 nm)/Mn_3_Sn (50 nm) device at 0.2 T (red), -0.2 T (blue) and zero field (green). Field-free current-induced switching cannot be observed for these devices.

**Section S10. Measurement of symmetry broken by nonlinear Hall effect**

The inversion symmetry breaking can be confirmed by the nonlinear Hall effect [12]. As a low-frequency AC current ($I_{\omega}$) is applied along the $x$($y$) direction, the second harmonic voltage ($V_{2\omega}$) is measured along the $y$($x)$ direction. We observe the nonlinear Hall effect in both the Mn_3_Sn (50 nm) and the reference Ru/Mn_3_Sn and Ru/Mn_3_Sn/Pt Hall-bar devices of the same size. However, the magnitude of the nonlinear Hall effect for the Mn_3_Sn device is much stronger than that of the reference samples. This suggests our Mn_3_Sn device without heavy metal layer has stronger inversion asymmetry, which probably originates from the boundaries between the grains and/or magnetic domains in the polycrystals. The strong inversion asymmetry can result in a large Rashba effect.


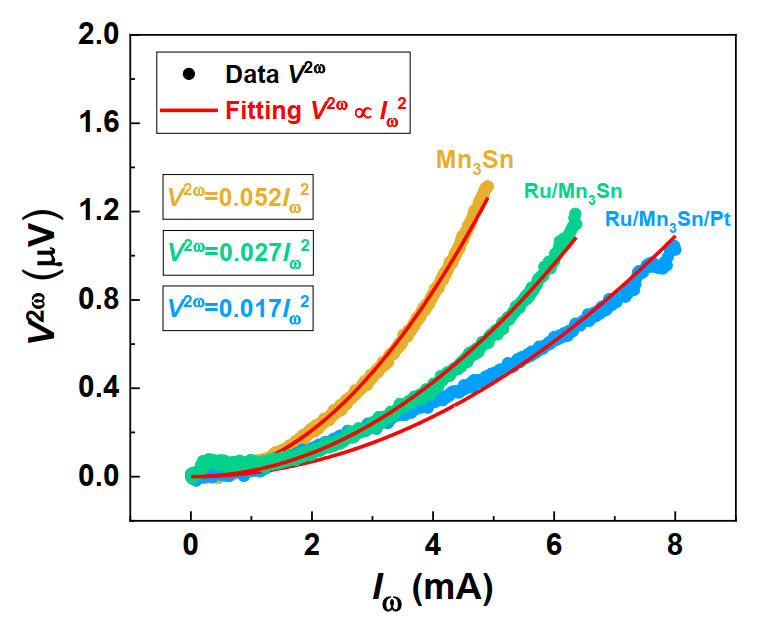


**Supplementary Fig. S9 | Nonlinear Hall effect of Mn_3_Sn and related heterostructures.** The second harmonic Hall voltage is quadratic to the amplitude of alternating current along the longitudinal direction, the quadratic coefficient of Mn_3_Sn (50 nm), Ru (3 nm)/Mn_3_Sn (50 nm), and Ru (3 nm)/Mn_3_Sn (50 nm)/Pt (6 nm) are 0.052, 0.027, 0.017 V/A^2^, respectively.

**Section S11. Estimate of Joule heating**

First, we measure the longitudinal resistivity $\rho_{xx}$ as a function of temperature (from 300 to 460 K) for the Mn_3_Sn (50 nm) device (Supplementary Fig. S10a). Then $\rho_{xx}$ of the sample is measured during the current pulse, ranging from 0 to 36 mA, at 300 K (Supplementary Fig. S10b). By comparing $\rho_{xx}$ during the current pulse to the measured temperature dependence of $\rho_{xx}$ without the current pulse in Supplementary Fig. S10a, the temperature is estimated to be increased by about 38 K due to the Joule heating induced by a 50-ms current pulse of 36 mA, as shown by the arrows in Supplementary Fig. S10b. Nevertheless, 338 K is still much lower than the Néel temperature of our Mn_3_Sn thin films ($\approx430 K$) (Fig. 1f and Supplementary Fig. S7). Therefore, we can rule out the dominance of the Joule heating in the observed switching.


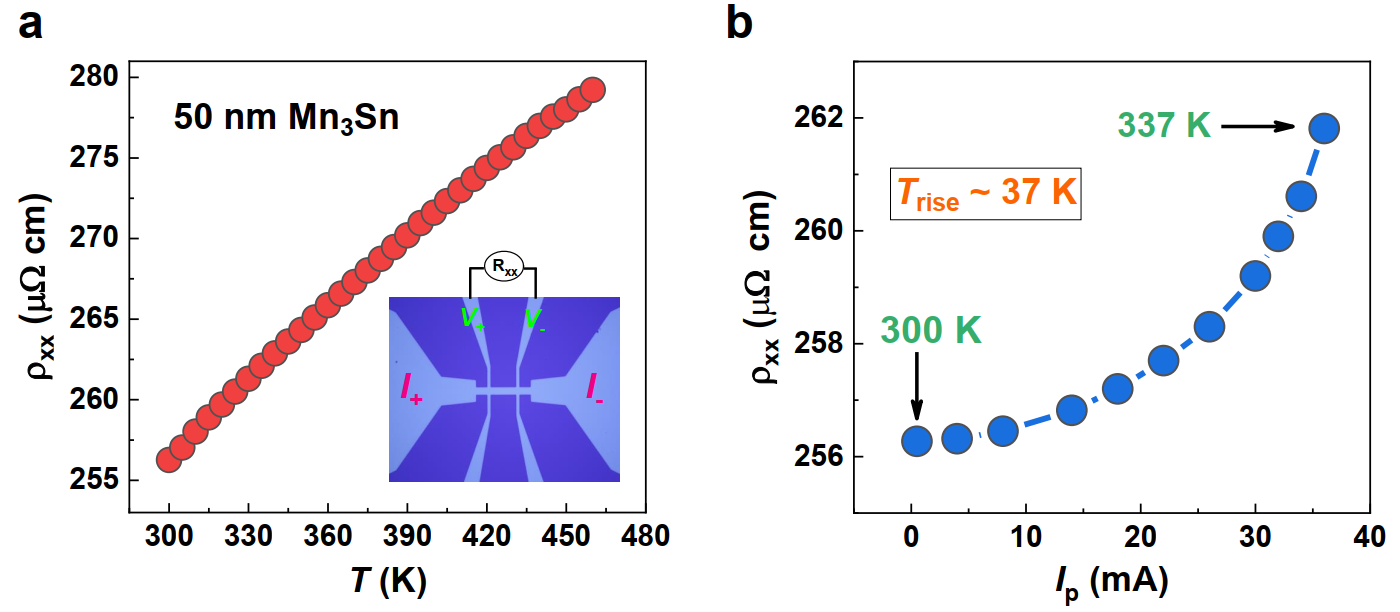


**Supplementary Fig. S10 | Evaluation of the Joule heating induced by the current pulse in the Mn_3_Sn (50 nm) device**. (a) Longitudinal resistivity $\rho_{xx}$ as a function of temperature. The inset shows the measurement setup. (b) Longitudinal resistivity $\rho_{xx}$ measured during the current pulse as a function of the current pulse magnitude $I_{p}$. The pulse width is fixed at 50 ms.

**Section S12. Current-induced antiferromagnetic states switching without external field at temperatures of 200K and 250K**


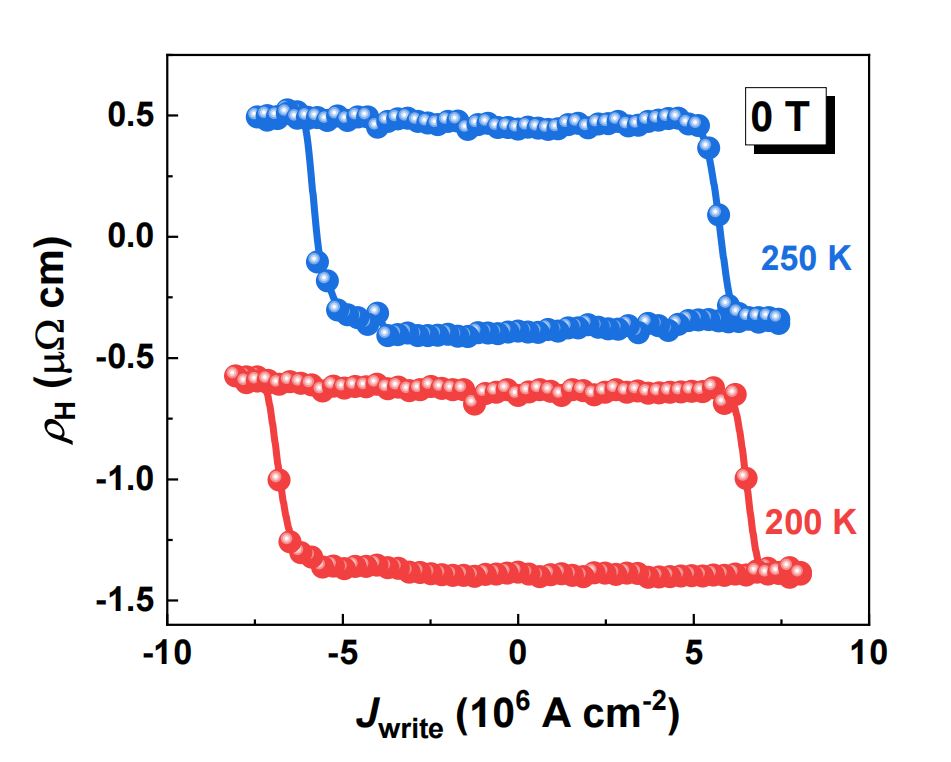


**Supplementary Fig. S11 | Anomalous Hall resistivity** $\boldsymbol{\rho}_{\mathbf{H}}$ **as a function of the writing current (with density of** $\boldsymbol{J}_{\mathbf{write}}$**) at zero field and 250 K (blue) and 200 K (red), as a readout of the** **current-induced antiferromagnetic states switching in the 50-nm-thick Mn_3_Sn device at zero magnetic field.** It is found that the $\rho_{H}-J_{\text{write}}$ loops display a similar switching behavior as that in Fig. 2b but with a slightly larger threshold current density at lower temperatures.

**Section S13. Symmetry analysis on why no switching in single crystal of Mn_3_Sn with inversion symmetry**

For Mn_3_Sn with an arbitrary magnetic structure, the nontrivial symmetry operators are $P$, $TC_{2y}$, and $TPC_{2y}$. If the electronic Hamiltonian follows the same symmetry, the deterministic switching of octupole is not achievable, for the macroscopic and microscopic reasons as follows.

Macroscopically, consider the following experimental device setup (see the figure below).


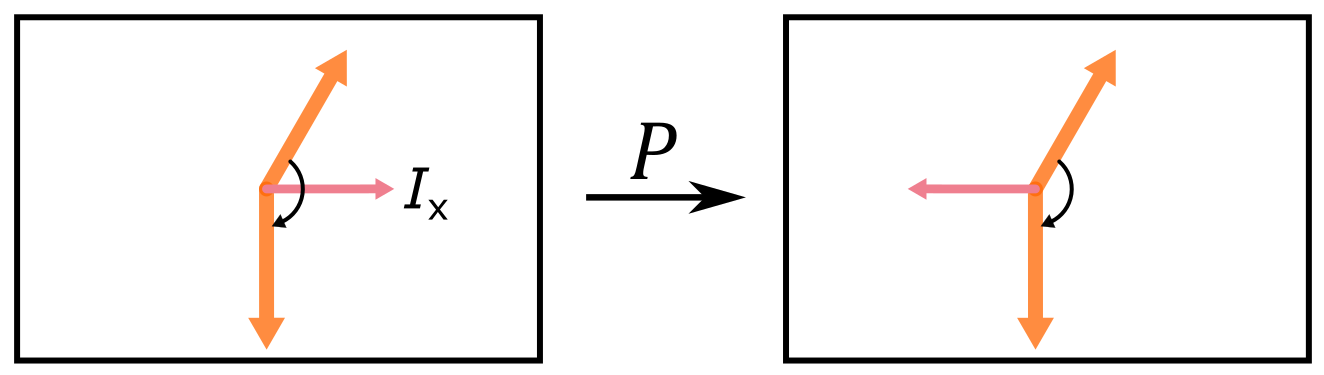


If a rightward current brings the initial octupole downward, with an inversion operation, the current, as a polar vector, turns to the opposite direction, but the octupole, as an axial vector, would not change. If our system preserves inversion symmetry, it is equivalent to the original system with an opposite current after the inversion operation, which means that an opposite current would bring the octupole to the same final state. Thus, there is no deterministic switching of the octupole if there is inversion symmetry.

Microscopically, consider the equations (Eqs. 3 and 4 in Methods) that we use to calculate the local spin accumulations on the sublattice.

$$\delta\left\langle\mathbf{s}_{a} \right\rangle_{d}=-\frac{e\hbar}{2}\tau E^{\beta}\sum_{n} \int\frac{d\mathbf{k}}{(2\pi)^{2}}\frac{\partial f}{\partial\varepsilon_{n}}\left\langle n,\mathbf{k} | \upsilon^{\beta} | n,\mathbf{k} \right\rangle\left\langle n,\mathbf{k} | \boldsymbol{\sigma}^{a} | n,\mathbf{k} \right\rangle,$$

$$\delta\left\langle\mathbf{s}_{a} \right\rangle_{od}=-\frac{e\hbar^{2}}{2}E^{\beta}\sum_{m\neq n} \int\frac{d\mathbf{k}}{(2\pi)^{2}}\left( f_{m}-f_{n} \right)\times\mathrm{Im} \left[ \frac{\left\langle m,\mathbf{k} | \upsilon^{\beta} | n,\mathbf{k} \right\rangle\left\langle n,\mathbf{k} | \boldsymbol{\sigma}^{a} | m,\mathbf{k} \right\rangle}{(\varepsilon_{m}-\varepsilon_{n})^{2}} \right].$$

$v^{\beta}$ is the velocity operator in the $\beta=\{x,y,z\}$ direction and $\boldsymbol{\sigma}^{a}$ is the local spin operator on site $a=\{1,2,3,1^{'},2^{'},3^{'}\}$ (see the figure below).


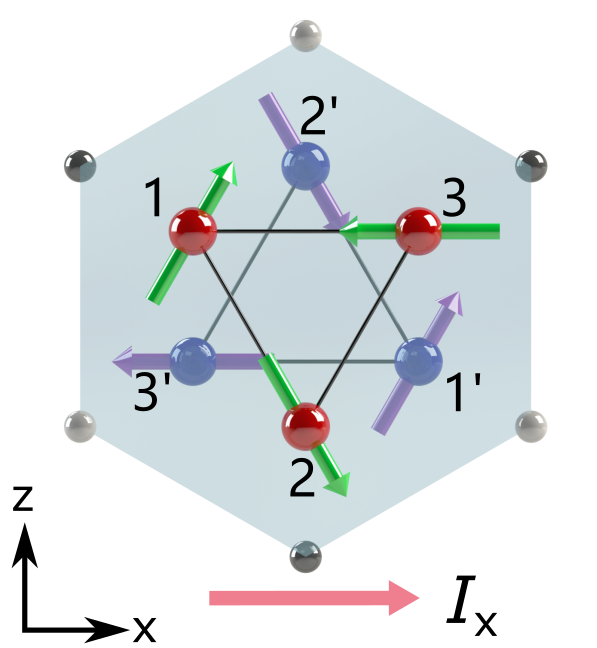


If our system preserves inversion symmetry, the equations can be rewritten as

$$\delta\left\langle\mathbf{s}_{a} \right\rangle_{d}=-\frac{e\hbar}{2}\tau E^{\beta}\sum_{n} \int\frac{d\mathbf{k}}{(2\pi)^{2}}\frac{\partial f}{\partial\varepsilon_{n}}\left\langle Pn,\mathbf{k} | \upsilon^{\beta} | Pn,\mathbf{k} \right\rangle\left\langle Pn,\mathbf{k} | \boldsymbol{\sigma}^{a} | Pn,\mathbf{k} \right\rangle$$

$$=-\frac{e\hbar}{2}\tau E^{\beta}\sum_{n} \int\frac{d\mathbf{k}}{(2\pi)^{2}}\frac{\partial f}{\partial\varepsilon_{n}}\left\langle n,\mathbf{k} | {P^{-1}\upsilon}^{\beta}P | n,\mathbf{k} \right\rangle\left\langle n,\mathbf{k} | {P^{-1}\boldsymbol{\sigma}}^{a}P | n,\mathbf{k} \right\rangle,$$

$$\delta\left\langle\mathbf{s}_{a} \right\rangle_{\mathrm{od}}=-\frac{e\hbar^{2}}{2}E^{\beta}\sum_{m\neq n} \int\frac{d\mathbf{k}}{(2\pi)^{2}}\times\left( f_{m}-f_{n} \right)\mathrm{Im} \left[ \frac{\left\langle Pm,\mathbf{k} | \upsilon^{\beta} | Pn,\mathbf{k} \right\rangle\left\langle Pn,\mathbf{k} | \boldsymbol{\sigma}^{a} | Pm,\mathbf{k} \right\rangle}{(\varepsilon_{m}-\varepsilon_{n})^{2}} \right]$$

$$-\frac{e\hbar^{2}}{2}E^{\beta}\sum_{m\neq n} \int\frac{d\mathbf{k}}{(2\pi)^{2}}\left( f_{m}-f_{n} \right)\times\mathrm{Im} \left[ \frac{\left\langle m,\mathbf{k} | P^{-1}\upsilon^{\beta}P | n,\mathbf{k} \right\rangle\left\langle n,\mathbf{k} | {P^{-1}\boldsymbol{\sigma}}^{a}P | m,\mathbf{k} \right\rangle}{(\varepsilon_{m}-\varepsilon_{n})^{2}} \right].$$

The velocity is a polar vector and should be reversed under the inversion operation. The local spin as an axial vector should be unchanged but the diagonal sites should be swapped, i.e., $1\leftrightarrow1^{'}$, $2\leftrightarrow2^{'}$, $3\leftrightarrow3^{'}$. In conclusion, for the unit cell in a system with inversion symmetry in the above figure, the current-induced local spin accumulations on the diagonal sites should be opposite, rotating the local moments in opposite directions. For the inverse triangular spin structure, the local moment on diagonal sites tends to align, so the opposite rotation tendencies induced by the opposite spin accumulations would cancel each other and thus there is no switching of the magnetic structure.

**Section S14. Symmetry analysis to justify the Rashba spin-orbit coupling in single crystal of Mn_3_Sn with broken inversion symmetry**

The inversion symmetry breaking leads to two consequences, the first is the Rashba spin-orbit coupling, the second is an energy difference between the $\{1,2,3\}$ and $\{1’,2’,3’\}$ kagome layers.

After introducing the Rashba spin-orbit coupling to the Hamiltonian, the non-trivial symmetry operators of the system with general magnetic structure reduce from $P$, $TC_{2y}$, $TPC_{2y}$ to $TC_{2y}$, where inversion symmetry and mirror-time reversal symmetry are broken. For the symmetry operator $TC_{2y}$, we have

$$\delta\left\langle\mathbf{s}_{a} \right\rangle_{d}=-\frac{e\hbar}{2}\tau E^{\beta}\sum_{n} \int\frac{d\mathbf{k}}{(2\pi)^{2}}\frac{\partial f}{\partial\varepsilon_{n}}\left\langle TC_{2y}n,\mathbf{k} | \upsilon^{\beta} | TC_{2y}n,\mathbf{k} \right\rangle\left\langle TC_{2y}n,\mathbf{k} | \boldsymbol{\sigma}^{a} | TC_{2y}n,\mathbf{k} \right\rangle$$

$$=-\frac{e\hbar}{2}\tau E^{\beta}\sum_{n} \int\frac{d\mathbf{k}}{(2\pi)^{2}}\frac{\partial f}{\partial\varepsilon_{n}}\left\langle n,\mathbf{k} | {{(TC_{2y})}^{-1}\upsilon}^{\beta}TC_{2y} | n,\mathbf{k} \right\rangle\left\langle n,\mathbf{k} | {{(TC_{2y})}^{-1}\boldsymbol{\sigma}}^{a}TC_{2y} | n,\mathbf{k} \right\rangle,$$

$$\delta\left\langle\mathbf{s}_{a} \right\rangle_{\mathrm{od}}=-\frac{e\hbar^{2}}{2}E^{\beta}\sum_{m\neq n} \int\frac{d\mathbf{k}}{(2\pi)^{2}}\left( f_{m}-f_{n} \right)\times\mathrm{Im} \left[ \frac{\left\langle TC_{2y}m,\mathbf{k} | \upsilon^{\beta} | TC_{2y}n,\mathbf{k} \right\rangle\left\langle TC_{2y}n,\mathbf{k} | \boldsymbol{\sigma}^{a} | TC_{2y}m,\mathbf{k} \right\rangle}{(\varepsilon_{m}-\varepsilon_{n})^{2}} \right]$$

$$-\frac{e\hbar^{2}}{2}E^{\beta}\sum_{m\neq n} \int\frac{d\mathbf{k}}{(2\pi)^{2}}\left( f_{m}-f_{n} \right)\times\mathrm{Im} \left[ \frac{\left\langle n,\mathbf{k} | {(TC_{2y})}^{-1}\upsilon^{\beta}TC_{2y} | m,\mathbf{k} \right\rangle\left\langle m,\mathbf{k} | {{(TC_{2y})}^{-1}\boldsymbol{\sigma}}^{a}TC_{2y} | n,\mathbf{k} \right\rangle}{(\varepsilon_{m}-\varepsilon_{n})^{2}} \right].$$

In our case the current is in the $x$ direction, so the velocity operator is unchanged under the symmetry operator $TC_{2y}$, i.e., ${(TC_{2y})}^{-1}v_{x}TC_{2y}=v_{x}$. The local spin in the kagome plane is also unchanged but the diagonal sites should be swapped, i.e., ${(TC_{2y})}^{-1}\sigma_{x,z}^{1,2,3}TC_{2y}=\sigma_{x,z}^{1^{'},2^{'},3^{'}}$. Thus, for the diagonal spin accumulation, we have $\delta{(s_{1,2,3}^{x,z})}_{d}=\delta{(s_{1^{'},2^{'},3^{'}}^{x,z})}_{d}$. However, note that the time-reversal operator changes the matrix elements to their conjugates, so for the off-diagonal spin accumulation, we have $\delta{(s_{1,2,3}^{x,z})}_{\mathrm{od}}=-\delta{(s_{1^{'},2^{'},3^{'}}^{x,z})}_{\mathrm{od}}$. The symmetry analysis shows that off-diagonal spin accumulation does not provide a switching torque to the magnetic structure. The above conclusion can be seen in Fig. S12 below.

As a result of the energy difference between the $\{1,2,3\}$ and $\{1’,2’,3’\}$ layers, the only non-trivial symmetry operators $TC_{2y}$ would no longer leave the system invariant and the conclusion above would not be valid anymore. Nevertheless, as we show in Fig. S13 below, a reasonable energy difference ($\Lambda=0.1t$, comparable with the Rashba spin-orbit coupling) would not cause much change to the conclusion. Specifically, the dash-dot lines in Fig. S13 shows that the off-diagonal (od) contribution is at least three orders smaller than the diagonal (d) contribution to the spin accumulations, and we have $\delta{(s_{1,2,3}^{x,z})}_{d}\approx\delta{(s_{1^{'},2^{'},3^{'}}^{x,z})}_{d}$ and $\delta{(s_{1,2,3}^{x,z})}_{\mathrm{od}}\approx-\delta{(s_{1^{'},2^{'},3^{'}}^{x,z})}_{\mathrm{od}}$ in the presence of a reasonable energy difference between the neighboring kagome layers resulted from the inversion symmetry breaking. Therefore, we assume $\Lambda=0$in the main text, to simply the illustration of the switching mechanism (otherwise, we need to show six Mn moments, instead of only three Mn moments).


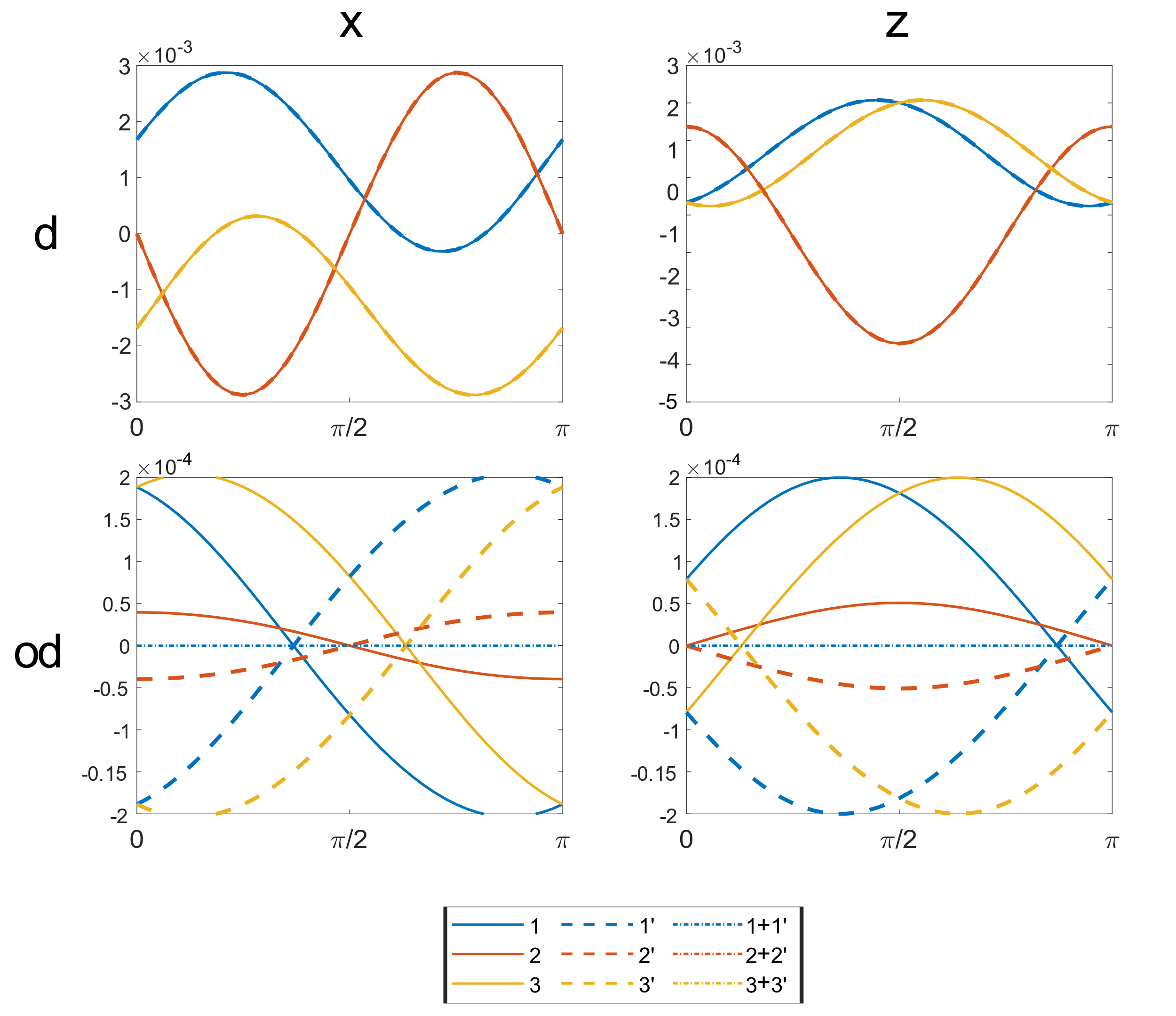


**Supplementary Fig. S12 |** Spin accumulations given by Eq. 3 and Eq. 4 of Methods for the system without an energy difference between the $\{1,2,3\}$ and $\{1’,2’,3’\}$ layers ($\Lambda=0$). Because of the the $TC_{2y}$ symmetry, it is revealed that $\delta{(s_{1,2,3}^{x,z})}_{d}=\delta{(s_{1^{'},2^{'},3^{'}}^{x,z})}_{d}$ and $\delta{(s_{1,2,3}^{x,z})}_{\mathrm{od}}=-\delta{(s_{1^{'},2^{'},3^{'}}^{x,z})}_{\mathrm{od}}$.


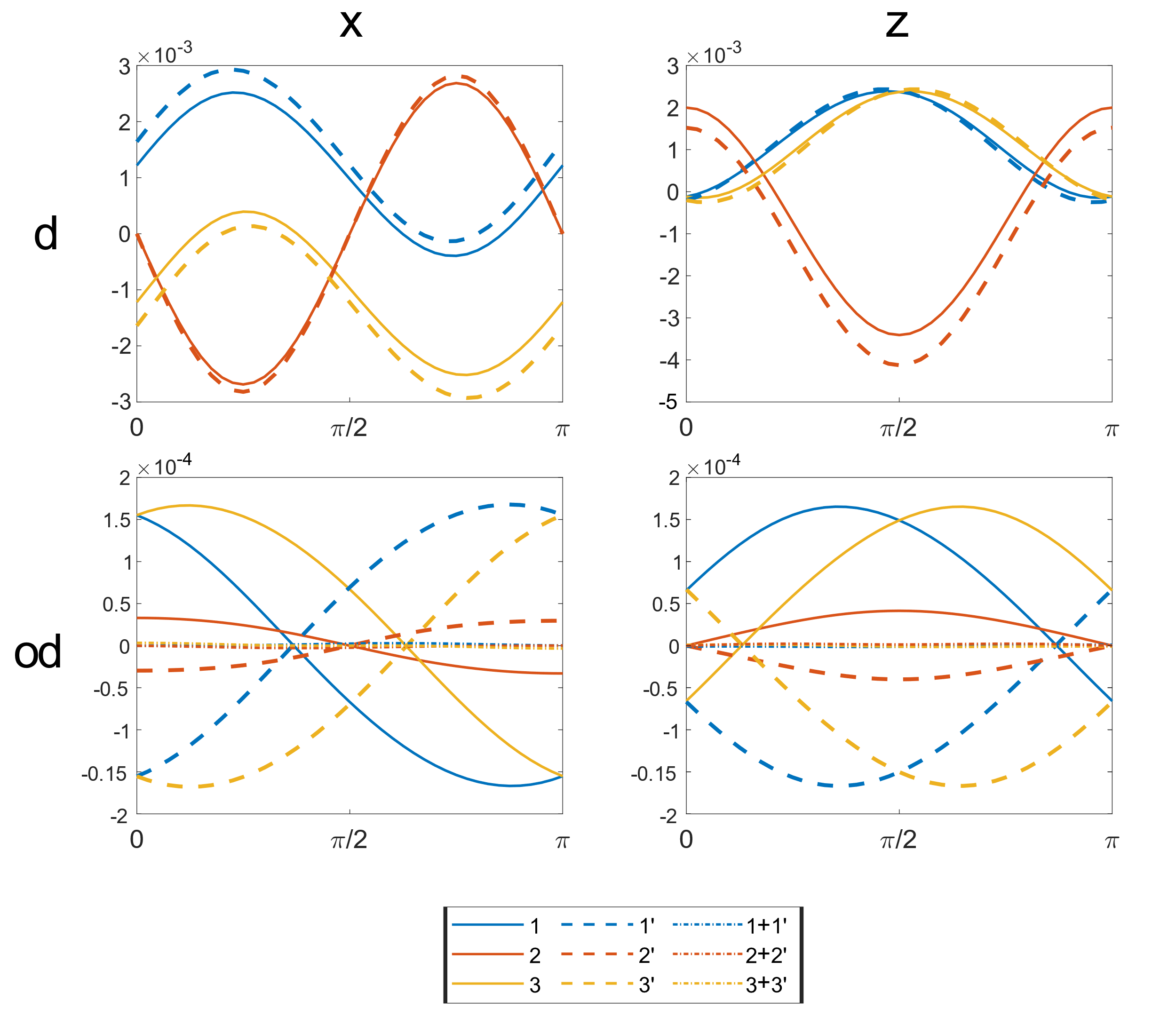


**Supplementary Fig. S13 |** Spin accumulations given by Eq. 3 and Eq. 4 of Methods for the system with a reasonable energy difference between the $\{1,2,3\}$ and $\{1’,2’,3’\}$ layers ($\Lambda=0.1t$, comparable with the Rashba spin-orbit coupling). $\delta{(s_{1,2,3}^{x,z})}_{d}=\delta{(s_{1^{'},2^{'},3^{'}}^{x,z})}_{d}$ and $\delta{(s_{1,2,3}^{x,z})}_{\mathrm{od}}=-\delta{(s_{1^{'},2^{'},3^{'}}^{x,z})}_{\mathrm{od}}$ are no longer valid since the energy difference breaks the $TC_{2y}$ symmetry. Nevertheless, the energy difference does not cause much change to the conclusion, i.e., we still have $\delta{(s_{1,2,3}^{x,z})}_{d}\approx\delta{(s_{1^{'},2^{'},3^{'}}^{x,z})}_{d}$, $\delta{(s_{1,2,3}^{x,z})}_{\mathrm{od}}\approx-\delta{(s_{1^{'},2^{'},3^{'}}^{x,z})}_{\mathrm{od}}$, and the off-diagonal (the dash-dot lines in the od row) is at least three orders smaller than the diagonal (d) contribution to the spin accumulations.

**Section S15. Calculation of octupole moment from magnetic structure**

The magnetic structure of Mn_3_Sn at room temperature is the so-called inverse triangular spin structure with space-inversion symmetry. The lowest rank of non-zero cluster multipole is 3, which is the octupole. For the $D_{6h}$ group of Mn_3_Sn, all the octupoles of different irreducible representations are

| Irreducible representation | Octupole |
| --- | --- |
| $A_{2g}$ | $T_{y}^{\alpha}\equiv{-M}_{30}$ |
| $E_{1g}$ | $T_{x}^{\gamma}\equiv\frac{1}{\sqrt{2}}\left( -M_{31}+M_{3-1} \right)$ $T_{z}^{\gamma}\equiv-\frac{i}{\sqrt{2}}\left( M_{31}+M_{3-1} \right)$ |
| $E_{2g}$ | $T_{xyz}\equiv\frac{i}{\sqrt{2}}\left( M_{32}-M_{3-2} \right)$ $T_{y}^{\beta}\equiv-\frac{1}{\sqrt{2}}\left( M_{32}+M_{3-2} \right)$ |
| $B_{2g}$ | $T_{x}^{\zeta}\equiv\frac{1}{\sqrt{2}}\left( -M_{33}+M_{3-3} \right)$ |
| $B_{1g}$ | $T_{z}^{\zeta}\equiv\frac{i}{\sqrt{2}}\left( M_{33}+M_{3-3} \right)$ |

For Mn_3_Sn with inverse triangular spin structure, the components of the octupole moment $O$ with a net macroscopic magnetization are given by [13]

$O_{x}\equiv T_{x}^{\gamma}=\frac{1}{\sqrt{2}}\left( -M_{31}+M_{3-1} \right)$,$O_{z}\equiv T_{z}^{\gamma}=-\frac{i}{\sqrt{2}}\left( M_{31}+M_{3-1} \right)$,

where a rank-$p$ magnetic polarization moment is defined as

$M_{pq}\equiv\frac{N_{\mathrm{atom}}^{u}}{N_{\mathrm{atom}}^{c}}\frac{1}{V}\sum_{\mu=1}^{N_{\mathrm{cluster}}} \sqrt{\frac{4\pi}{2p+1}}\sum_{i=1}^{N_{\mathrm{atom}}^{(\mu)}} \boldsymbol{m}_{a}\cdot\nabla_{i}(\left| \boldsymbol{R}_{i} \right|^{p}Y_{pq}{(\theta_{i},\varphi_{i})}^{*})$,

and $N_{\mathrm{atom}}^{u}$ is the number of atoms in the magnetic unit cell, $N_{\mathrm{atom}}^{c}=\sum_{\mu} N_{\mathrm{atom}}^{(\mu)}$ is the total number of atoms in all clusters, $V$ is the volume of magnetic unit cell and $N_{\mathrm{cluster}}$ is the number of clusters in the unit cell, $N_{atom}^{(\mu)}$ is the number of atoms of the $\mu$*-*th cluster (there are two type of clusters for Mn_3_Sn, as shown in the figure below), $\boldsymbol{m}_{a}$ is a magnetic moment on the $a$-th atom, $\nabla_{a} \equiv\partial/\partial\boldsymbol{R}_{a}$, $\boldsymbol{R}_{a}\equiv(X_{a},Y_{a},Z_{a})$ is the position of the ith atom, $Y_{pq}$ are the spherical harmonics, and $|\boldsymbol{R}_{a}|,\theta_{a}$, and $\varphi_{a}$ are the distance, polar angle, and azimuthal angle, respectively, of the $a$-th atom.


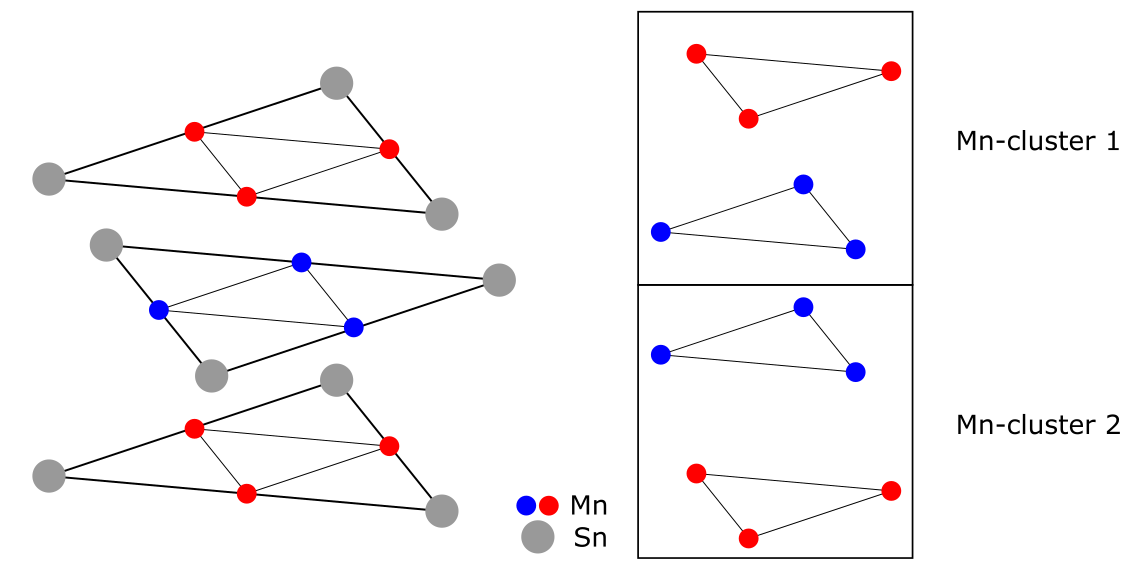


Each of the following magnetic structures gives an octupole with same magnitude in the $x$ and $z$ directions, respectively.


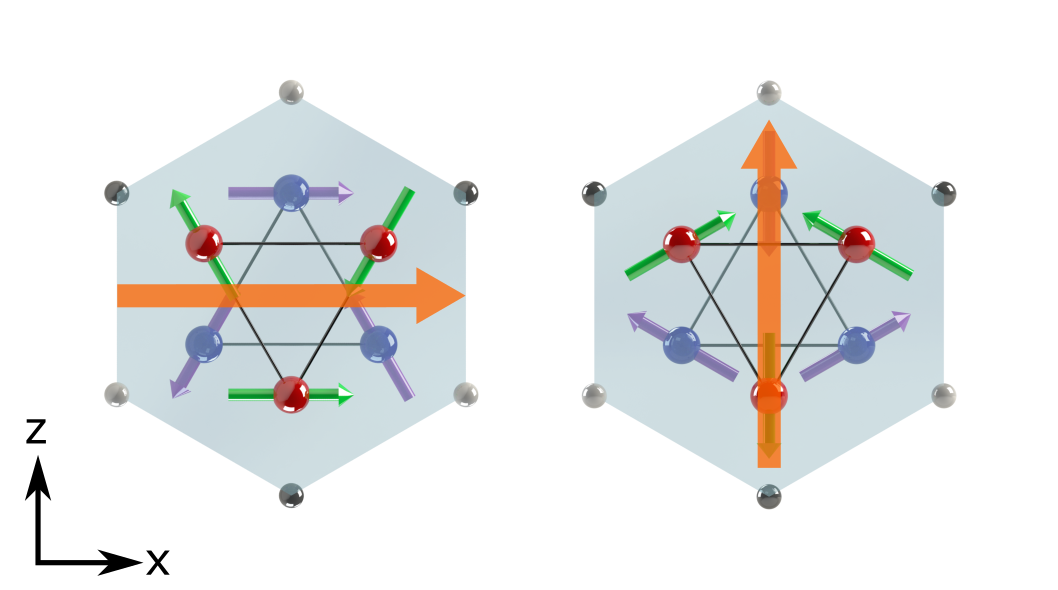


For the rest of the magnetic structures with arbitrary directions, they can be represented by the linear combination of these two magnetic structures. If we label the direction with the polar angle $\varphi_{2}$ of Mn 2 (red sphere at the bottom) on the $x-z$ plane $\mathbf{(m}_{i2}=\left( \left( \mathbf{m}_{i2} \right)_{x},\left( \mathbf{m}_{i2} \right)_{z} \right)=(sin\varphi_{2},\cos\varphi_{2}))$, a magnetic structure labeled by the octupole components can be given as:

$\left\{ \boldsymbol{m}_{i} \right\}=\cos\varphi_{2}\left\{ \boldsymbol{m}_{i} \right\}^{x}-\sin\varphi_{2}\left\{ \boldsymbol{m}_{i} \right\}^{z}$,$\hat{O}_{x}=\sin\varphi_{2}={(\mathbf{m}_{i2})}_{x},\hat{O}_{z}=-\cos\varphi_{2}=-{(\mathbf{m}_{i2})}_{z}$,

where $\left\{ \boldsymbol{m}_{i} \right\}^{x}$ and $\left\{ \boldsymbol{m}_{i} \right\}^{z}$ represents the magnetic structure of AFM1 and AFM2. The above analysis shows that the direction of octupole can be determined by the direction of Mn 2.

**Section S16. Effects of spin-orbit coupling, magnetic-structure parameters, and initial states on simulation results**

We show more simulation results for different key parameters, including the Rashba spin-orbit coupling $\lambda_{R}$ (in Eq. 2 of Methods) and those for the magnetic structure $J_{m},D, K$ (in Eq. 1 of Methods). The results show that stronger Rashba spin-orbit coupling leads to an enhanced spin accumulation, faster switching, and smaller critical current. A larger $K/(J_{m},D)$ provides stronger torque that pulls the magnetic structure to one of the six stable positions of the octupole. To achieve the switching, the torque from the external current needs to overcome the stable-position-favored torque from the magnetic structure.


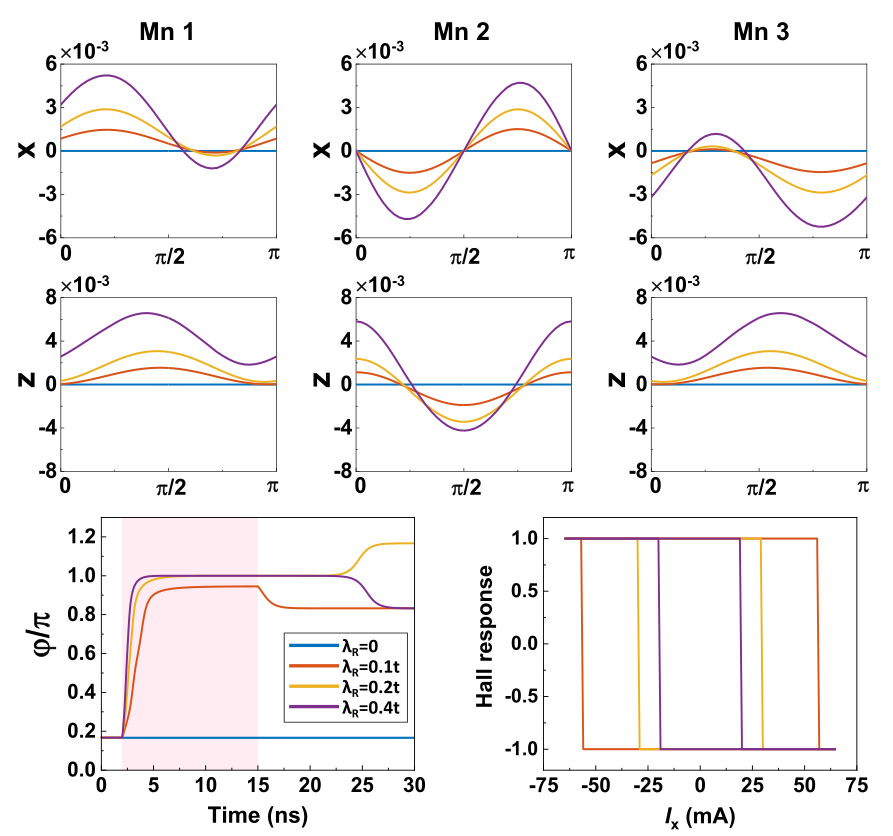


**Supplementary Fig. S14 | Top 6 panels:** The same as Fig. 4c in the main text, but for different Rashba spin-orbit coupling $\lambda_{R}$, i.e., the simulated spin accumulations along the $x$ and $z$ axes on all three Mn sublattices (Mn 1, Mn 2, Mn 3) on a single Mn_3_Sn kagome layer, as a function of the polarization angle of Mn 2 measured from the $x$ axis on the $x-z$ plane, in units of $s_{0}=I_{x}m^{*}/2n_{3D}ea_{L}$, where $m^{*}$ is the effective mass of electron, $n_{3D}$ is the carrier density, $e$ is the elementary charge, and $a_{L}$ is the lattice constant on the kagome layer. Only the diagonal (“d”) linear-response formula (Eq. 3 in Methods) is shown, because the off-diagonal part yields compensated spin accumulations thus does not contribute to the switching (see Section S14). The spin accumulations on each Mn atom increase as $\lambda_{R}$ increases from $0$ to $0.4t$, where $t$ is the kinetic term in the s-d model (Eq. 2 in Methods). More spin accumulations lead to stronger torques acting on the magnetic structure. $\lambda_{R}=0$ corresponds to the system without Rashba type spin-orbit coupling, which preserves inversion symmetry and mirror-time reversal symmetry, therefore has zero-spin accumulation on all Mn atoms. **Bottom left panel:** The simulated switching of the magnetic structure (octupole) for different $\lambda_{R}$ with $I_{x}=180mA$. With this injected current, $\lambda_{R}=0.1t, 0.2t$ and $0.4t$ all lead to successful switching with a slight difference. $\lambda_{R}=0.1t$ is not able to fully polarize the octupole to $\varphi=\pi$ while $\lambda_{R}=0.2t$ and $\lambda_{R}=0.4t$ fully polarize the octupole. The switching at $\lambda_{R}=0.4t$ is the fastest. **Bottom right panel:** The $I_{x}$-hysteresis and critical current for different $\lambda_{R}$. The critical current decreases with increasing spin-orbit coupling $\lambda_{R}$, specifically, $I_{\mathrm{critical}}^{0.1t}=57mA$, $I_{\mathrm{critical}}^{0.2t}=30mA$ and $I_{\mathrm{critical}}^{0.4t}=20mA$. The behavior is consistent with the expectation that the stronger spin-orbit coupling $\lambda_{R}$, the larger magnitude of spin accumulations and more efficient switching.


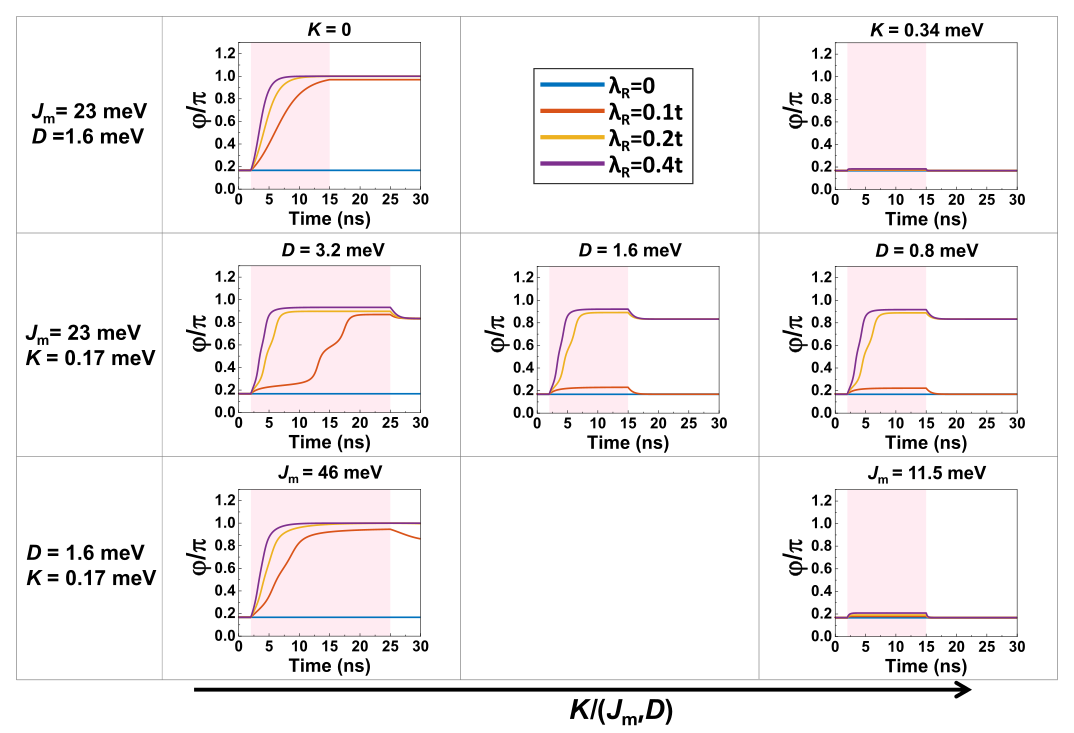


**Supplementary Fig. S15 |** The switching simulation for different magnetic-structure parameters at $I_{x}=50mA$, so that $I_{\mathrm{critical}}^{0.1t}>I_{x}>I_{\mathrm{critical}}^{0.2t}>I_{\mathrm{critical}}^{0.4t}$. **Center panel.** Here, the parameters of the magnetic structure $J_{m}=23meV, D=1.6meV, K=0.17meV$. Other panels show the deviations from the center column. According to the previous study on the Mn_3_Sn magnetic structure [14], $J_{m}$ and $D$ stabilize the inverse 120-degree triangular magnetic structure with a $U(1)$ symmetry, which is broken by $K$, creating a six-fold symmetry with 6 stable positions of the (octupole) magnetic structure with a small deviation from the inverse 120-degree magnetic structure. A larger $K/(J_{m},D)$ comes with a larger deviation and larger torque that pulls the structure to one of the stable positions. To achieve the switching, the torque from the external current needs to overcome the stable-position-favored torque from the magnetic structure. **Top left panel.** If $K=0$ or equivalently $K/{(J}_{m},D)=0$, an arbitrarily small $\lambda_{R}$ can switch the magnetic structure to the direction perpendicular to the current ($\varphi=\pi$) with only a difference in the switching speed from the spin-orbit coupling strength. **Top right panel.** For $K$ large enough, the torque from the current cannot overcome the stable-position-favored torque from the magnetic structure even for $\lambda_{R}=0.2t$ and $\lambda_{R}=0.4t$. **Center right panel.** Reducing $D$ by half does not cause observable change to the results, because in this case the stiffness of the inverse 120-degree triangular magnetic structure is not changed a lot by reducing $D$. **Center left panel.** If $D$ is doubled, the case of $\lambda_{R}=0.1t$ also reaches the goal of switching because in this case the stiffness of the magnetic structure is increased by doubling $D$. **Bottom right panel.** If $J_{m}$ is small enough, there is no switching. **Bottom left panel.** If $J_{m}$ is strong enough, there is always switching. In all the panels, a higher $\lambda_{R}$ always causes a faster switching and a further deviation from the stable position in a failed switching. Also, a smaller $K/(J_{m},D)$ comes with a slower relaxation after injecting the current.

We also show simulation results for all stable positions as initial states with the same injected current. As shown in Fig. S16, the final state of the magnetic structure is not related to the initial states but only determined by the direction of current. Here we show the simulated results for stable positions as initial states, but the conclusion is also valid for non-stable positions as initial states.


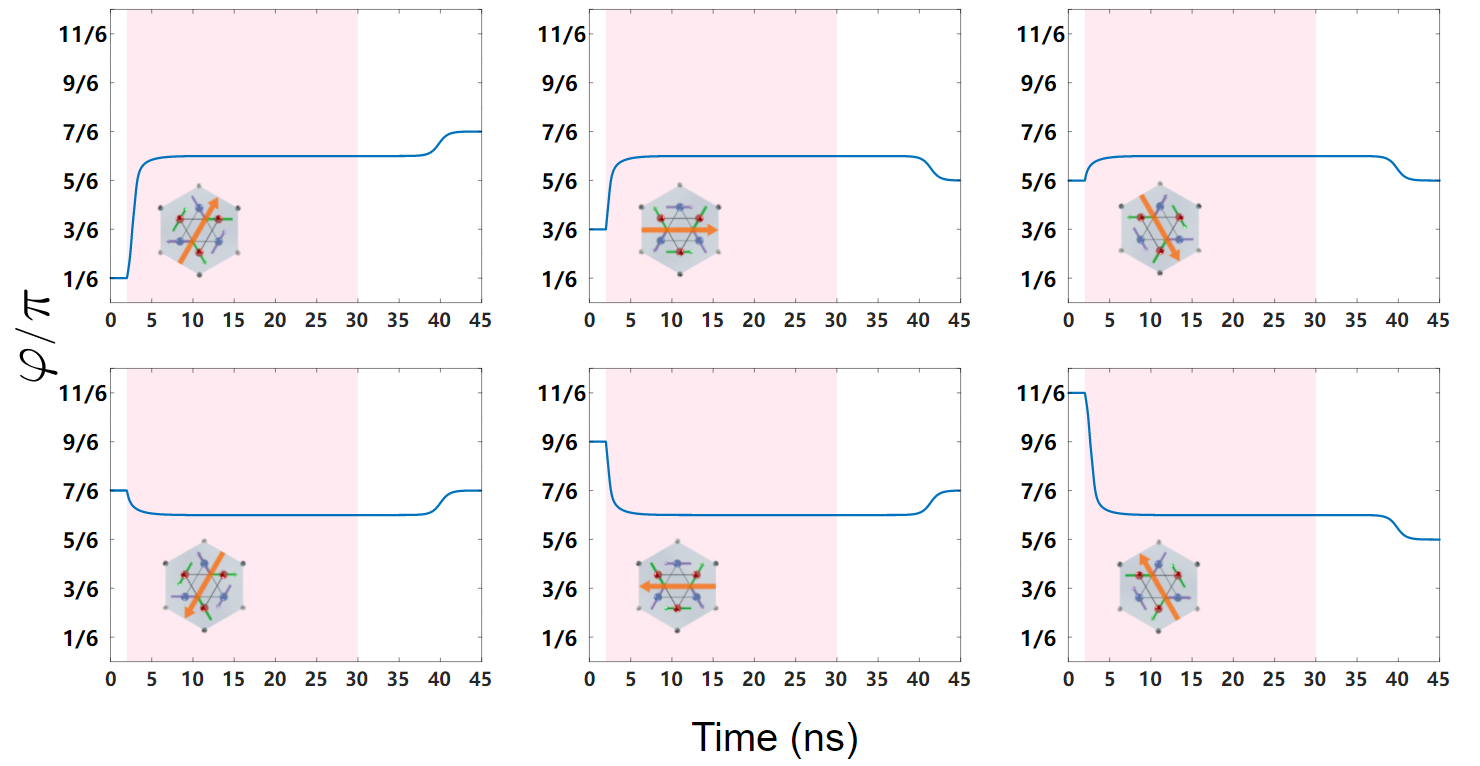


**Supplementary Fig. S16 |** The simulated octupole angle $\varphi$ (measured from the $z$ direction on the $x-z$ plane) as a function of time with current well above critical current ($I_{x}=180mA$) for different initial states. As we can see from the figures, different initial states are polarized to the same final state by the injected current and then randomly relax to the nearest stable positions. The figure shows that our switching mechanism is not influenced by the initial state.

**Section S17. The elements of the 12×12 matrix of the s-d model Hamiltonian**

$H=\left( \begin{matrix} H_{11} & H_{12} & H_{13} \\ H_{12}^{\dagger} & H_{22} & H_{23} \\ H_{13}^{\dagger} & H_{23}^{\dagger} & H_{33} \end{matrix} \right)$,

where

$H_{11}=\left( \begin{matrix} \Lambda& J_{sd}e^{-i(\varphi-\frac{\pi}{2})} & t & i\lambda_{R}(-\frac{1}{2\sqrt{3}}-\frac{i}{2}) \\ J_{sd}e^{i(\varphi-\frac{\pi}{2})} & \Lambda& i\lambda_{R}(-\frac{1}{2\sqrt{3}}+\frac{i}{2}) & t \\ t & i\lambda_{R}(\frac{1}{2\sqrt{3}}+\frac{i}{2}) & 0 & J_{sd}e^{-i(\varphi+\frac{\pi}{6})} \\ -i\lambda_{R}(-\frac{1}{2\sqrt{3}}+\frac{i}{2}) & t & J_{sd}e^{i(\varphi+\frac{\pi}{6})} & 0 \end{matrix} \right)$,

$H_{22}=\left( \begin{matrix} \Lambda& J_{sd}e^{-i(\varphi+\frac{5\pi}{6})} & t & i\lambda_{R}(-\frac{1}{2\sqrt{3}}+\frac{i}{2}) \\ J_{sd}e^{i(\varphi+\frac{5\pi}{6})} & \Lambda& i\lambda_{R}(-\frac{1}{2\sqrt{3}}-\frac{i}{2}) & t \\ t & -i\lambda_{R}(-\frac{1}{2\sqrt{3}}+\frac{i}{2}) & 0 & J_{sd}e^{-i(\varphi-\frac{\pi}{2})} \\ -i\lambda_{R}(-\frac{1}{2\sqrt{3}}-\frac{i}{2}) & t & J_{sd}e^{i(\varphi-\frac{\pi}{2})} & 0 \end{matrix} \right)$,

$H_{33}=\left( \begin{matrix} \Lambda& J_{sd}e^{-i(\varphi+\frac{\pi}{6})} & t & i\lambda_{R}(\frac{1}{\sqrt{3}}) \\ J_{sd}e^{i(\varphi+\frac{\pi}{6})} & \Lambda& i\lambda_{R}(\frac{1}{\sqrt{3}}) & t \\ t & i\lambda_{R}(-\frac{1}{\sqrt{3}}) & 0 & J_{sd}e^{-i(\varphi+\frac{5\pi}{6})} \\ i\lambda_{R}(-\frac{1}{\sqrt{3}}) & t & J_{sd}e^{i(\varphi+\frac{5\pi}{6})} & 0 \end{matrix} \right)$,

$H_{12}=\left( \begin{matrix} t\left( 1+e^{i\boldsymbol{k}\cdot\boldsymbol{a}_{2}} \right) & H_{12}^{12} & 0 & 0 \\ H_{12}^{21} & t\left( 1+e^{i\boldsymbol{k}\cdot\boldsymbol{a}_{2}} \right) & 0 & 0 \\ t & i\lambda_{R}\left( -\frac{1}{\sqrt{3}} \right) & t\left( 1+e^{i\boldsymbol{k}\cdot\boldsymbol{(a}_{2}-\boldsymbol{a}_{1})} \right) & H_{12}^{34} \\ i\lambda_{R}\left( -\frac{1}{\sqrt{3}} \right) & t & H_{12}^{43} & t\left( 1+e^{i\boldsymbol{k}\cdot\boldsymbol{(a}_{2}-\boldsymbol{a}_{1})} \right) \end{matrix} \right)$,

where

$H_{12}^{12}=i\lambda_{R}\left[ \left( -\frac{\sqrt{3}}{2}-\frac{i}{2} \right)+\left( \frac{\sqrt{3}}{2}+\frac{i}{2} \right)e^{i\boldsymbol{k}\cdot\boldsymbol{a}_{2}} \right]$,

$H_{12}^{21}=i\lambda_{R}\left[ \left( -\frac{\sqrt{3}}{2}+\frac{i}{2} \right)+\left( \frac{\sqrt{3}}{2}-\frac{i}{2} \right)e^{i\boldsymbol{k}\cdot\boldsymbol{a}_{2}} \right]$,

$H_{12}^{34}=i\lambda_{R}\left[ \left( -\frac{\sqrt{3}}{2}+\frac{i}{2} \right)+\left( \frac{\sqrt{3}}{2}-\frac{i}{2} \right)e^{-i\boldsymbol{k}\cdot\left( \boldsymbol{a}_{1}-\boldsymbol{a}_{2} \right)} \right]$,

$H_{12}^{43}=i\lambda_{R}\left[ \left( -\frac{\sqrt{3}}{2}-\frac{i}{2} \right)+\left( \frac{\sqrt{3}}{2}+\frac{i}{2} \right)e^{-i\boldsymbol{k}\cdot\left( \boldsymbol{a}_{1}-\boldsymbol{a}_{2} \right)} \right]$,

$H_{13}=\left( \begin{matrix} H_{13}^{11} & H_{13}^{12} & t & -i\lambda_{R}(\frac{1}{2\sqrt{3}}-\frac{i}{2}) \\ H_{13}^{21} & H_{13}^{22} & -i\lambda_{R}(\frac{1}{2\sqrt{3}}+\frac{i}{2}) & t \\ 0 & 0 & H_{13}^{33} & H_{13}^{34} \\ 0 & 0 & H_{13}^{43} & H_{13}^{44} \end{matrix} \right)$,

where

$H_{13}^{11}=t(1+e^{i\boldsymbol{k}\cdot\boldsymbol{(a}_{2}-\boldsymbol{a}_{1})})$, $H_{13}^{22}=t(1+e^{i\boldsymbol{k}\cdot\boldsymbol{(a}_{2}-\boldsymbol{a}_{1})})$,

$H_{13}^{33}=t(1+e^{-i\boldsymbol{k}\cdot\boldsymbol{a}_{1}})$, $H_{13}^{44}=t(1+e^{-i\boldsymbol{k}\cdot\boldsymbol{a}_{1}})$,

$H_{13}^{12}=-i\lambda_{R}[\left( \frac{\sqrt{3}}{2}-\frac{i}{2} \right)+\left( -\frac{\sqrt{3}}{2}+\frac{i}{2} \right)e^{-i\boldsymbol{k}\cdot(\boldsymbol{a}_{1}-\boldsymbol{a}_{2})}]$,

$H_{13}^{21}=-i\lambda_{R}[\left( \frac{\sqrt{3}}{2}+\frac{i}{2} \right)+\left( -\frac{\sqrt{3}}{2}-\frac{i}{2} \right)e^{-i\boldsymbol{k}\cdot(\boldsymbol{a}_{1}-\boldsymbol{a}_{2})}]$,

$H_{13}^{34}=-i\lambda_{R}(-i+ie^{-i\boldsymbol{k}\cdot\boldsymbol{a}_{1}})$, $H_{13}^{43}=-i\lambda_{R}(i-ie^{-i\boldsymbol{k}\cdot\boldsymbol{a}_{1}})$,

$H_{23}=\left( \begin{matrix} t(1+e^{-i\boldsymbol{k}\cdot\boldsymbol{a}_{1}}) & i\lambda_{R}(i-ie^{-i\boldsymbol{k}\cdot\boldsymbol{a}_{1}}) & 0 & 0 \\ i\lambda_{R}(-i+ie^{-i\boldsymbol{k}\cdot\boldsymbol{a}_{1}}) & t(1+e^{-i\boldsymbol{k}\cdot\boldsymbol{a}_{1}}) & 0 & 0 \\ t & i\lambda_{R}(\frac{1}{2\sqrt{3}}+\frac{i}{2}) & t(1+e^{-i\boldsymbol{k}\cdot\boldsymbol{a}_{2}}) & H_{23}^{34} \\ i\lambda_{R}(\frac{1}{2\sqrt{3}}-\frac{i}{2}) & t & H_{23}^{43} & t(1+e^{-i\boldsymbol{k}\cdot\boldsymbol{a}_{2}}) \end{matrix} \right)$,

where

$H_{23}^{34}=i\lambda_{R}[\left( \frac{\sqrt{3}}{2}+\frac{i}{2} \right)+\left( -\frac{\sqrt{3}}{2}-\frac{i}{2} \right)e^{-i\boldsymbol{k}\cdot\boldsymbol{a}_{2}}$,

$H_{23}^{43}=i\lambda_{R}[\left( \frac{\sqrt{3}}{2}-\frac{i}{2} \right)+\left( -\frac{\sqrt{3}}{2}+\frac{i}{2} \right)e^{-i\boldsymbol{k}\cdot\boldsymbol{a}_{2}}$.

**References:**

1. Tsai H, Higo T, Kondou K et al. Electrical manipulation of a topological antiferromagnetic state. Nature 2020;580:608–13.

2. DuttaGupta S, Kurenkov A, Tretiakov OA et al. Spin-orbit torque switching of an antiferromagnetic metallic heterostructure. Nat Commun 2020;11:5715.

3. Manchon A, Železný J, Miron IM et al. Current-induced spin-orbit torques in ferromagnetic and antiferromagnetic systems. Rev Mod Phys 2019;91:035004.

4. Kimata M, Chen H, Kondou K et al. Magnetic and magnetic inverse spin Hall effects in a non-collinear antiferromagnet. Nature 2019;565:627–30.

5. Patterson AL. The Scherrer Formula for X-Ray Particle Size Determination. Phys Rev 1939;56:978–82.

6. Khan A, Toufiq AM, Tariq F et al. Influence of Fe doping on the structural, optical and thermal properties of α-MnO_2_ nanowires. Mater Res Express 2019;6:065043.

7. Holder CF, Schaak RE. Tutorial on Powder X-ray Diffraction for Characterizing Nanoscale Materials. ACS Nano 2019;13:7359–65.

8. Higo T, Qu D, Li Y et al. Anomalous Hall effect in thin films of the Weyl antiferromagnet Mn_3_Sn. Appl Phys Lett 2018;113:202402.

9. Nandy S, Sharma G, Taraphder A et al. Chiral Anomaly as the Origin of the Planar Hall Effect in Weyl Semimetals. Phys Rev Lett 2017;119:176804.

10. Son DT, Spivak BZ. Chiral anomaly and classical negative magnetoresistance of Weyl metals. Phys Rev B 2013;88:104412.

11. Nakatsuji S, Kiyohara N, Higo T. Large anomalous Hall effect in a non-collinear antiferromagnet at room temperature. Nature 2015;527:212–5.

12. Ma Q, Xu S-Y, Shen H et al. Observation of the nonlinear Hall effect under time-reversal-symmetric conditions. Nature 2019;565:337–42.

13. Suzuki MT, Koretsune T, Ochi M et al. Cluster multipole theory for anomalous Hall effect in antiferromagnets. Phys Rev B 2017;95:094406.

14. Liu J, Balents L. Anomalous Hall Effect and Topological Defects in Antiferromagnetic Weyl Semimetals: Mn_3_Sn/Ge. Phys Rev Lett 2017;119:087202.
